# The importance of counterion association in the calculated binding constants of Ca^2+^-aminopolycarboxylate complexes

**DOI:** 10.1039/d5ra07375h

**Published:** 2026-03-18

**Authors:** Mojgan Heshmat, Pavlo Kostetskyy, Guanna Li, Daan S. van Es

**Affiliations:** a Wageningen Food & Biobased Research Bornse Weilanden 9 6708 WG Wageningen The Netherlands mojgan.heshmat@wur.nl daan.vanes@wur.nl; b Archer Daniels Midland Company 1001 N Brush College Road Decatur IL 62521 USA; c Biobased Chemistry & Technology, Wageningen University Bornse Weilanden 9 Wageningen 6708 WG The Netherlands

## Abstract

There exist a number of chemical reactions and processes that have elevated concentrations of metal ions both in solution and as part of a precipitate. To reduce the undesirable effects of metal cations and improve the performance of the active materials in various chemical systems, the application of chelators is required. To this end, different types of chelators have been developed with high affinity towards various metal cations. However, many of the most effective chelators used are limited in biodegradability and could have an impact on environmental pollution. Hence, development of highly efficacious biodegradable alternatives with high affinity for metal cations is an emerging challenge. Electronic structure calculations can assist in predicting new bio-sourced and biodegradable chelators by estimating the binding affinity of metal ion-chelator systems in aqueous solution. In this work, using a set of benchmark aminopolycarboxylate ligands, we calculate binding affinities for a number of chelator-ion systems and investigate the effect of including counterion association on the calculated binding constant. It was found that the system charge neutrality significantly improves the correlation between calculated and experimental values. Furthermore, the influence of conformational flexibility of the chelator structure and pH of the aqueous solution were addressed. The discussed modification in calculation of the log *K* values considering the counterion association currently serves as a prediction basis and a potential design tool toward new bio-based chelators for applications in industry.

## Abstract 2

In this work, using a set of benchmark aminopolycarboxylate ligands, we calculate binding affinities for a number of chelator-ion systems and investigate the effect of including counterion Na^+^ association on the calculated binding constant.

## Graphical abstract

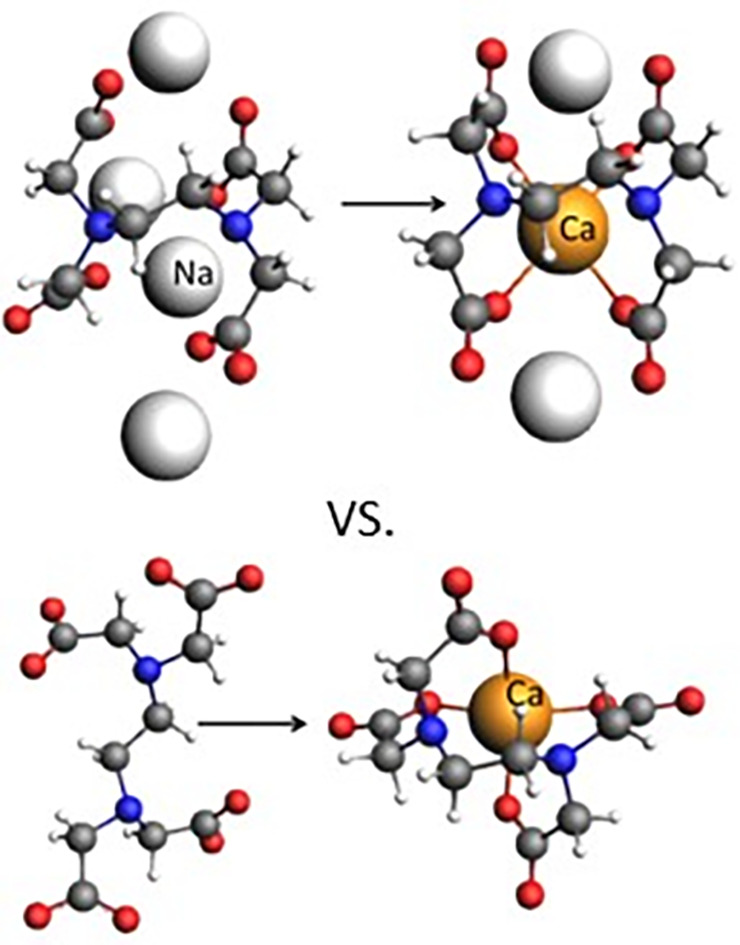

## 1 Introduction

The use of chelators is required in many industrial processes and consumer applications, *e.g.* home and personal care products, paper and textile manufacturing, to mitigate the undesirable effects of various metal ions such as Ca^2+^ or Mg^2+^.^1,2^ For example, in home and personal care products, calcium and magnesium cations that are present in tap water (water hardness) can produce scale and reduce the performance of detergents in multiple industrial and domestic processes such as laundering or dishwashing. To reduce water hardness, phosphonates such as (1-hydroxyethane-1,1-diyl)bis(phosphonic acid) (HEDP) and diethylenetriamine penta (methylene phosphonic acid) (DTPMP) may be used. Aminopolycarboxylates (APCs) are another class of industrially relevant chelators, often commercially supplied in the form of their alkali metal salts. Well known examples include ethylenediaminetetraacetic acid (EDTA) and diethylenetriaminepentaacetic acid (DTPA) used as sequestering agents due to their high affinity for Ca^2+^.^3^ However, due to the limited biodegradability and other negative environmental effects of these widely used chelators, there is a demand for new, fully renewable and readily biodegradable alternatives.^4–7^ While some commercial aminopolycarboxylic acids (MGDA, GLDA, EDDS *etc.*) are biodegradable, their technical performance is lacking compared to phosphonates. Therefore, there is a need for new, renewable, and biodegradable chelators with high affinities for specific metal ions.

Theoretical *ex-ante* predictions based on the stability constants of complex formation between metal cations and ligands are a valuable tool in identifying the potential of biobased and biodegradable alternatives for commercially available chelators. For chelating agents, the strength of the metal ion-ligand coordination bond in aqueous solution is of importance. The stability of a chelated metal complex is expressed by the stability constant *K*, which is the equilibrium constant for the formation of a complex in solution. Experimentally there are various methods to determine the stability constant *K* and for commercially applied chelating agents, these are often known.^8–11^ Many theoretical studies in this area addressed various approaches to improve the calculated values with respect to the experimental ones. To this end, effective parametrization methods using empirical correction parameters were introduced to correct for omitted energetic contributions to the calculated metal–ligand complexation energies. The defined parametrizations and corrections may closely correlate and be specific to the ligand and metal electronic characteristics as well as structural properties and may vary from one metal–ligand complex to another.^12–17^ This parameterization was done by introducing either one term energy correction for each metal or an additional correction for each ligand.^12,13^ To use DFT calculations as a predictive tool for the development of new biobased chelator molecules, a theoretical method is required that produces log *K* values that are close to the experimental ones in terms of absolute values and trends observed. According to the literature, the calculated log *K* values so far show large differences with respect to the experimentally measured ones, which is comparable to estimates of molecular acid/base properties such as pKa.^18–20^ Overestimation of the binding constant has been reported by Laasonen and co-workers. However, the impact of counterions such as Na^+^ (in addition to the metal cation of interest) association with the chelator-ion system that can physically exist in the experimental environment has not been clearly considered in these studies. In this work we calculate the binding constant values for different benchmark chelators to investigate the effect of including counterion association that significantly improves the correlation between calculated and experimental values without addition of extra parameters and corrections in formulation of the binding constant. According to the literature, it was observed that the formation constants of the Na^+^ complexes with mono-ligand stoichiometry (ML) linearly increase with the number of charges on the aminopolycarboxylate ligands. This indicates an ionic association between Na^+^ and anionic carboxylate ligands.^21^ To validate the effect of such association, we focus on three different ways of calculation of log *K*, *i.e.*, (i) including implicit water only, (ii) including implicit water + explicit water molecules, (iii) including implicit water only + counterions association. For explicit solvation we included six water molecules forming H-bonds with the ligand anions and for the counterion we added Na^+^ cations to completely balance the negative charge of the ligand anions. In addition, in this work, the influence of pH and conformational flexibility of the ligand–metal complex on the calculated binding constants is addressed. Isodesmic reactions, where the relative stability of various metal complexes in metal-exchange reactions are calculated, have been widely reported in literature for calculating relative reaction energies due to maximizing the cancellation of errors from the DFT method and from continuum solvent models.^22–28^ In order to calculate the stability constant values of new biobased chelators with a high degree of confidence, the computational method was validated using a set of known benchmark chelators.

## 2 Computational details

All geometries have been optimized using the Gaussian 16 package^29^ and verified to have zero imaginary frequencies. The calculations were carried out with the B3LYP exchange–correlation functional plus D3BJ dispersion correction^30–33^ and the triple-zeta plus additional polarization function basis set, 6-311G**. The Gibbs free-energies were calculated at 298 K and 1 atm in the solution phase, using the self-consistent continuum approximation (with the default PCM parameterization) of water. The linear transit scan to find the barrier of rotation has been performed using the ADF program package^34,35^ at BLYP-D3 TZP level of theory starting from and ending at fully optimized geometries.

## 3 Results and discussion

**Fig. 1:**
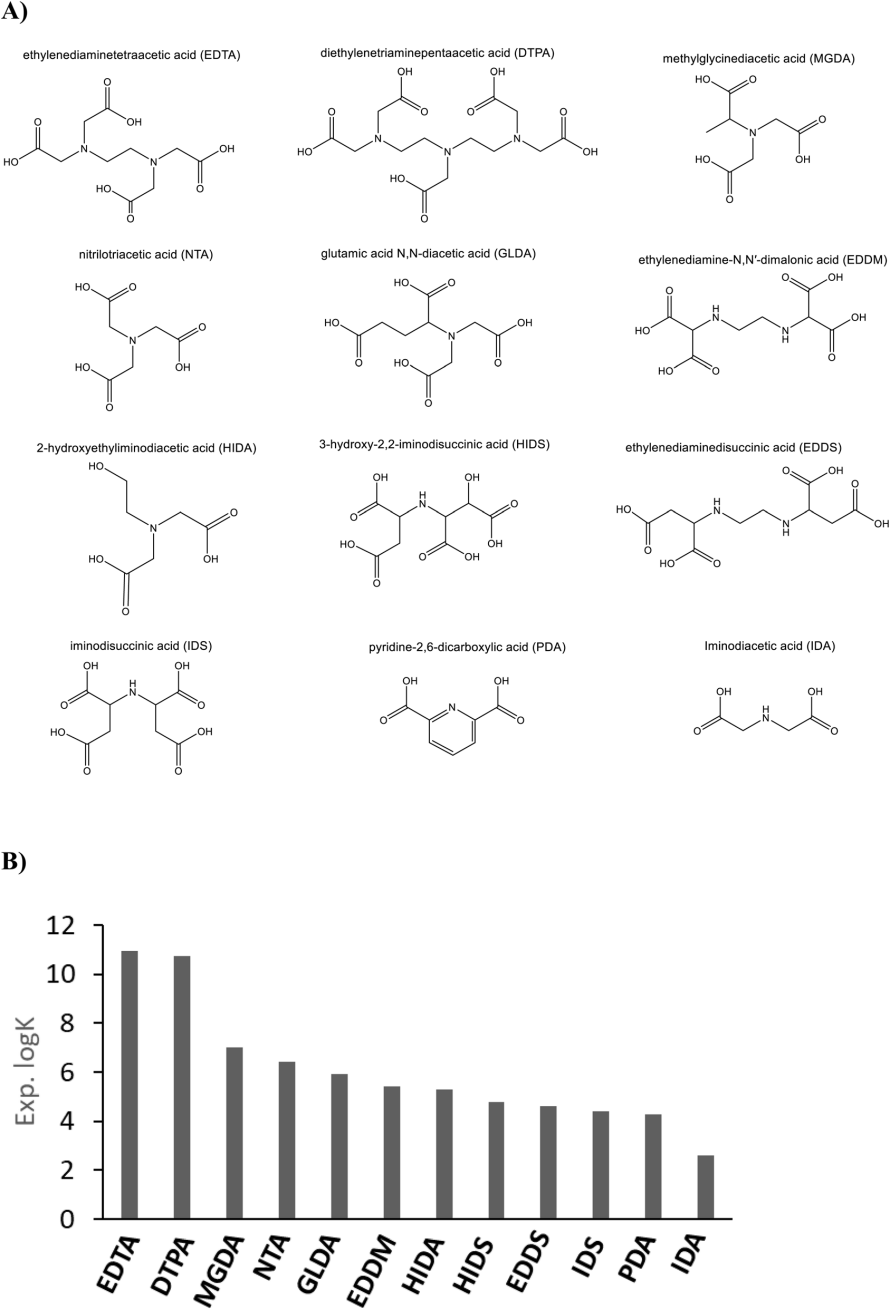
Schematic representation of the experimentally investigated chelators: (A) the structure of the selected benchmark chelators, please note that all the COOH groups are considered deprotonated in this work. (B) The measured log *K* values (binding constants measured at 25 °C and *µ* = 0.1 M) in order of decreasing binding affinity *vs.* Ca^2+^ based on literature reported data.

**Scheme 1:**
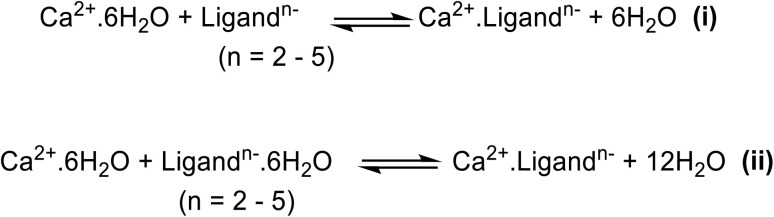
The reactions used for the calculation of the binding constant of the cation chelation including method (i) only implicit solvation and method (ii) explicit and implicit solvation for ligand molecules. The geometries for this reaction were fully optimized at B3LYP/D3BJ level of theory with 6-311G** basis set.

In Fig. 1A the molecular structures of the aminopolycarboxylic acids considered in this work are shown, while in Fig. 1B a comparison is made between the experimentally measured binding constants (log *K*) with respect to Ca^2+^ cation for this series.^3^ As can be seen in Fig. 1B, there is a clear correlation between several structural characteristics and the measured binding constants. Based on the observed experimental log *K* values, the most significant contributing factors, that lead to strong chelation, are the number of carboxylate and tertiary amine groups, as well as the length and flexibility of the carbon chains connecting these coordinating groups.^36–38^ Anionic carboxylate groups cause electronic charge transfer to the Ca^2+^ and result in strong covalent bond formation (chelation). We note that tertiary amines make stronger coordination than secondary amines, according to the experimental log *K* values (due to a more available donating lone pair). For example, the chelators with the highest Ca^2+^ affinity, EDTA and DTPA, have the highest number of tertiary amine (two and three respectively), and carboxylate groups (four and five respectively), as well as very short connecting chains (only one CH_2_), resulting in conformationally stable five or six membered rings in the complex. One possible theoretical model for obtaining the stability constants of metal ion-chelator complexes is based on the calculated Δ*G* of the reaction between hydrated calcium cation in the form of an octahedral molecular complex of Ca^2+^(H_2_O)_6_ and the chelator anion to produce the Ca^2+^ chelator molecular complex and release water molecules (as shown in reaction ii, Scheme 1). In this reaction the chelator anion is considered as an explicitly solvated species with *e.g.* six water molecules as the first solvation shell that form H-bonds with carboxylate anions. According to the literature, application of explicit and implicit solvation improves the calculated binding constant.^39–41^

Calculation of log *K* (*K* is the equilibrium constant for the reactions in Scheme 1) is based on eqn (1)–(4):

1 Δ*G*_reaction_ = ∑*G*(product molecules) − ∑*G*(reactant molecules)

2 log *K*_i/ii_ = −Δ*G*_i/ii_/*RT*(ln 10)

log *K*_exp_≡log *K*_calcd_

3 log *K*_calcd_ = log *K*_i_ − 6 log[H_2_O] for reaction i

4 log *K*_calcd_ = log *K*_ii_ − 12 log[H_2_O] for reaction ii

We note that Δ*G*(prd.) is the summation of Gibbs free energies of all molecular species on the product side (including H_2_O molecules) and Δ*G*(react.) is summation of the Gibbs free energies of all molecular species on the reactant side. In reactions i and ii the ligand anion is considered as a fully deprotonated species with −2 to −5 negative charge (depending on the number of carboxylate groups) in the calculations. The free energies calculated with Gaussian using ideal-gas partition functions are referenced to 1 atm, whereas the experimentally determined *K* in solution is referenced to a concentration of 1 M. Hence, the free energy of standard-state change must be considered. This term has no effect when the number of moles of reactants and products is the same, however, this is not the case in reactions i and ii due to formation of 6 and 12 H_2_O molecules, respectively. The calculated conversion constant for Δ*G*(1 atm → 1 M) is *ca.* 1.89 kcal mol^−1^ (details in SI). Furthermore, in order to convert the calculated equilibrium constants by method i and ii (log *K*_i_ and log *K*_ii_) into an equilibrium constant that can be better compared with the experimental one, we performed the calculations shown in eqn (3) and (4), which subtract 6 log[H_2_O] and 12 log[H_2_O] from log *K* of equations i and ii, respectively. The concentration of water is a constant, ∼55.5 M, and therefore log[H_2_O] is also a constant (∼1.74), which is identical for all cases. Including 1 atm to 1 M correction, the values of log *K*_calcd_ were calculated and reported in Tables S8 and S9 in SI, for reaction i and ii, respectively (details in SI).

**Fig. 2:**
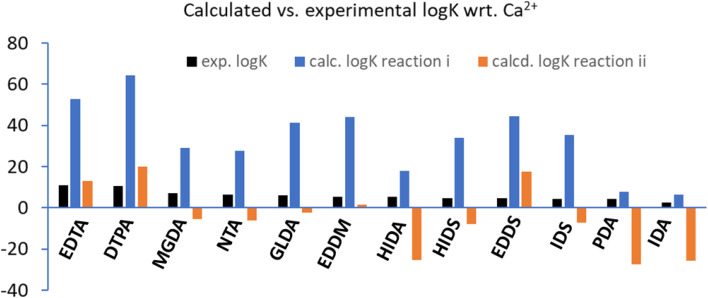
The calculated log *K* values based on reaction i and ii*vs.* the experimentally measured values. The descending order of log *K*_exp_ from EDTA to IDA is not observed for the calculated ones.

In solution, reaction ii means that an exchange between water molecules and ligand occurs for the calcium cation. In Fig. 2 we show the calculated log *K*_calcd_ values based on the eqn (1)–(4) and reactions i and ii (Scheme 1), for each ligand of Fig. 1A. As can be seen in Fig. 2, the calculated values (log *K*_i_) are distributed for some of the chelators (*e.g.* EDTA, DTPA, EDDM, GLDA) between 40–60 units, and the descending order of the experimental log *K* that can be observed from EDTA to IDA is not observed for the calculated values and they are distributed randomly. The values of log *K*_ii_ are reduced with respect to log *K*_i_, however, some are not realistic (negative values). Hence, the coefficient of determination (*R*^2^) between calculated (based on the reaction i and ii) and measured log *K* values is low. The particularities of the molecular structures of the chelators are not expressed in terms of an impact on the calculated values *versus* the experimental values. There are two issues that need to be addressed: first, a very large difference in log *K* values obtained by calculations and experiments and second, a poor correlation between experimental and calculated values is observed. In the next section the influence of ligand electronic structure and negative charge on the calculated log *K* values in correlation to the experiment are discussed to address these problems.

To modify log *K*_calcd_ values obtained by reactions of Scheme 1, there have been systematic efforts to explore the selectivity of metal binding by DFT approach. These efforts remarkably improved estimation of the metal ion complexation constant with various ligands.^12,32–34^ However, to the best of our knowledge, association of counterion that is naturally present in the reaction environment was not considered in these attempts, hence, this work.

### 3.1 Effect of the electronic structure of the ligand on the complexation to Ca^2+^

**Fig. 3:**
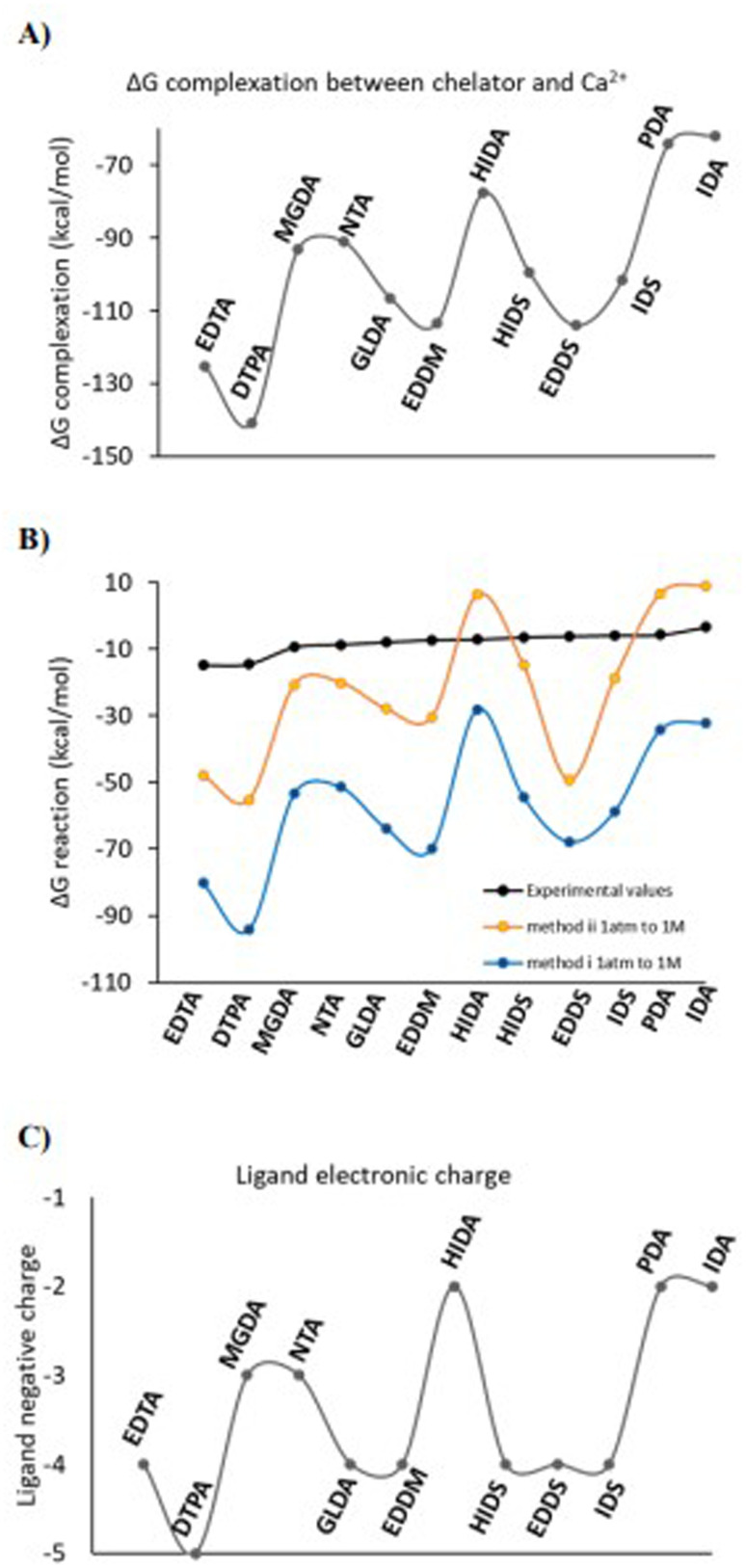
A) variation of the Δ*G* of the molecular complex formation between ligands and Ca^2+^ (the difference between Gibbs free energies of Ca^2+^⋯chelators molecular complex and free Ca^2+^ and chelator) no explicit water molecules is considered, (B) variation of the Δ*G* for the reactions i without explicit water molecules (blue) and with explicitly hydrated ligand (in orange) corresponding to the Scheme 1 and log *K* values in Fig. 2 in comparison to experimental Δ*G* values calculated from the available experimental log *K* values (black), (C) variation of the negative electronic charge of the ligand. In all calculations the implicit solvation using water was included. The lines guide eyes and show the trends.

In Fig. 3A the variation of the Δ*G* of complexation between chelators and Ca^2+^ (with a 1 : 1 ratio) for each ligand is shown. The Δ*G* of complexation is the difference between Gibbs free energy of Ca^2+^-chelator molecular complex and the summation of the Gibbs free energies of free Ca^2+^ and free chelator, *i.e.*, the free energy of reaction Ca^2+^ + chelator^*n*−^ → Ca-chelator^(*n*−2)−^. Variation of the Δ*G* of reactions i and ii (Scheme 1) is shown in 3B with explicitly hydrated chelators (in orange, method ii) in comparison to the Δ*G* without explicitly hydrated chelators (in blue, method i) and the Δ*G* experimental obtained from measured log *K* values (in black). The negative electronic charge of each ligand is depicted in 3C assuming complete deprotonation of the carboxylic acid groups. Secondary amine and hydroxyl groups are assumed to remain protonated (the lines are there to guide the eyes). Fig. 3B indicates that inclusion of explicit water molecules interacting with the chelator structure (H-Bonds) substantially improves the Δ*G* values with respect to only implicit solvation (orange *vs.* blue plots). We note that implicit solvation using water was applied in all calculations. As can be seen in Fig. 3A–C a rather similar pattern for all three graphs is observed, which is in principle similar to the pattern of variation of the negative electronic charge on the ligand anion (3C). This observation indicates that the negative electronic charge on the ligand controls the order of the Δ*G* of complexation between Ca^2+^ and chelator as well as the Δ*G* of reactions in Scheme 1 for the entire ligand series. Hence, the calculated log *K* values for reaction i and ii are under the influence of the negative electronic charge of the ligand and not the inherent structural characteristics of the ligand. However, as already shown in Fig. 1, the structural particularities of the ligands are determining for the experimental log *K* and the observed trend of the log *K*_exp_. These observations identify that reaction i and ii (and consequently the pattern of the calculations) is under the control of the very strong electrostatic interaction between the entirely negatively charged anion and Ca^2+^, which overshadows the specific but more subtle effects of chelator structure and functional groups. This indicates that the negative charge on the ligand should be balanced in such a way that it diminishes the very strong electrostatic interaction between metal cation and the ligand anion. This is a valid assumption since a polyanion cannot be solely stabilized by water in a real solution. Accordingly, non-stabilized multiple anionic sites will cause significant electrostatic repulsion and strain in the chelator, while in reality polyanions are dissolved in a stabilizing medium and are always neutralized by counterions.

For method i with anionic free ligands, we performed the conformer rotamer ensemble sampling tool (CREST) code,^42–45^ to find the lowest conformers of the free flexible ligand structures with various dihedral angles. The input structures for CREST were fully optimized geometries of ligands at B3LYP-D3BJ level of theory. Moreover, the ligand structures were calculated with including the explicit water molecules equal to the number of functional groups, *i.e.* 5H_2_O for DTPA, 4H_2_O for EDTA, EDDM, EDDS, HIDS, GLDA, IDS, 3H_2_O for MGDA and NTA, and 2H_2_O for, HIDA, IDA and PDA. The results are reported in Table S15 and Fig. S3 in SI. The correlation between calculation and experiment slightly improved with respect to the case with six water molecules for all ligands, *i.e.*, 0.07 units for *R*^2^ (0.5779 *vs.* 0.5002).

### 3.2 Counterion association

**Scheme 2:**
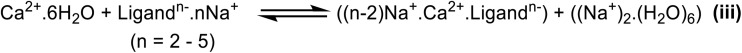
The modified reaction used for the calculation of the binding constant (method iii) which contains Na^+^ counterions (including implicit solvation but no explicit water molecules). We note that (Na^+^)_2_·6H_2_O is a single cluster of two Na^+^ cations and six water molecules.

**Fig. 4:**
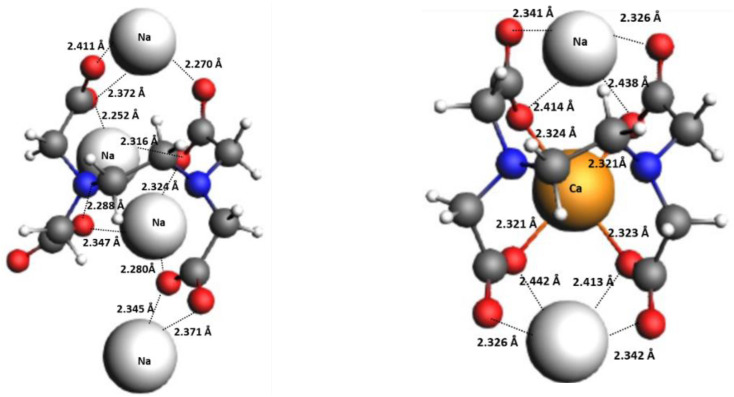
The optimized molecular structures for EDTA in reaction iii (Scheme 2), *i.e.*, associated with Na^+^ (left) and EDTA-Ca^2+^-Na^+^ (right). No explicit water solvent molecules were included for the chelator (method iii).

Based on these illustrations, compensation of the negative charge on the ligand is a key step before chelation. To this end, we developed an improved model system by association of the counterions to the chelator anion to realistically overcome the influence of strong electrostatic interaction between Ca^2+^ and ligand anion. Furthermore, according to the literature, the measurements of log *K* were performed on the ligands salts.^3^ Hence, we added extra Na^+^ cations to the reactions of Scheme 1 and complexed them to the ligand structure to balance the entire negative charge of the ligand before complexation to Ca^2+^ and reach to a fully neutralized ligand⋯Na^+^ salt.^46^ The modified reaction is shown in Scheme 2 (reaction iii). In fact, reaction iii represents an exchange between Na^+^ and Ca^2+^ cations with H_2_O and the chelator. To investigate the direct influence of the Na^+^ counterion association, explicit water molecules are not included in the chelator molecular structures in reaction iii. As example, Fig. 4 shows the molecular structures of free EDTA anion associated with Na^+^ and the molecular complex of EDTA-Ca^2+^-Na^+^. The advantage of applying reaction iii in the calculation of log *K* is twofold. First, full neutralization of the negative charge of the anion that compensates the large electrostatic interaction between Ca^2+^ and ligand (also within the polyanionic ligand). And second, involving the thermodynamic effects of exchange between Ca^2+^ and Na^+^ cations with H_2_O and the ligand in the overall calculated Δ*G* of the reaction. Full neutralization of the negative charge reduces the electrostatic interactions which is confirmed by Energy Decomposition Analysis (EDA)^47–49^ results as well as comparison of the complexation energies between Ca^2+^ and ligand^*n*−^*versus* Ca^2+^ and Ligand^*n*−^Na^+^_(*n*−2)_ (reported in Tables S5–S7). Involvement of exchange between cations in a reaction was investigated in previous studies as it maximizes the cancellation of systematic errors inherent to the level of theory and in the calculation of interactions between the continuum solvent and the solute, which can vary significantly with the net electric charge of the solute. As example, calculation of relative energies, thermodynamic constants (p*K*_a_ values), or the stability of metal complexes using cations exchange were frequently addressed in the literature.^24–27^

**Fig. 5:**
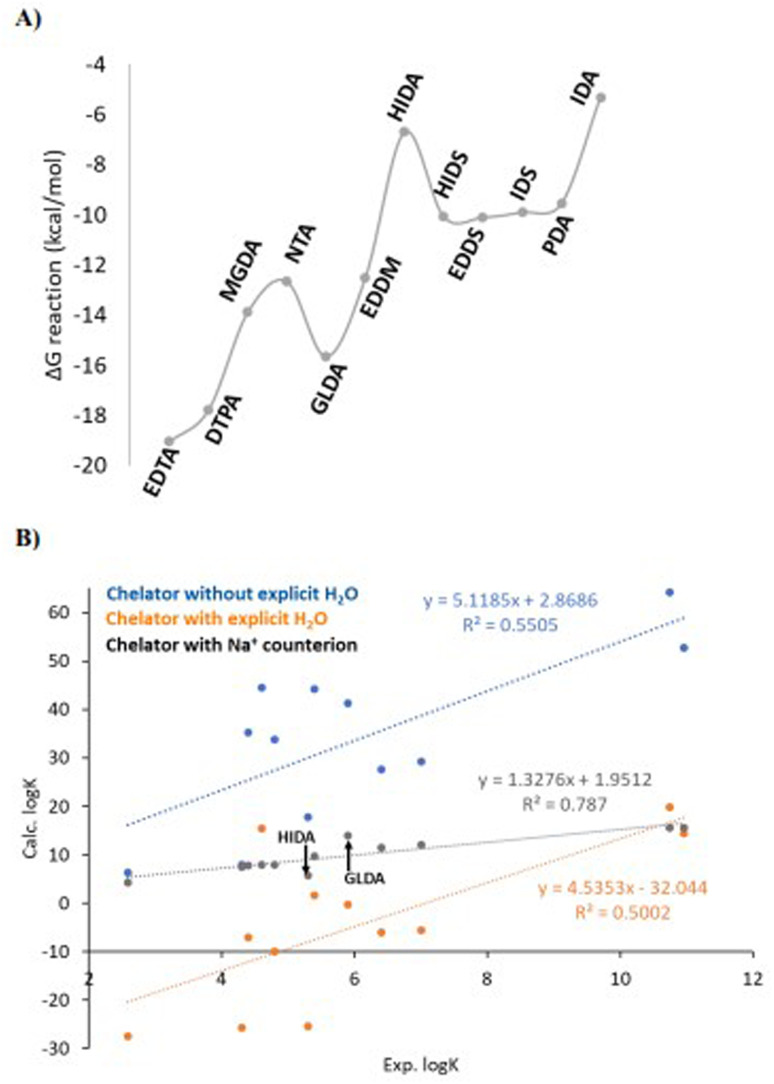
(A) the calculated free energies for the reaction iii (Scheme 2) considering the Na^+^ counterion association (correlated to the grey line in B). (B) variation of the calculated *vs.* experimental log *K* values for different models, *i.e.*, chelators without explicit water molecules (blue, case i), chelators including explicit water molecules (orange, case ii) and chelators considering the Na^+^ counter ion (grey, case iii) and no explicit water molecules.

Fig. 5A shows the calculated Δ*G* based on reaction iii (Scheme 2) for the ligand series considered in Fig. 1. Fig. 5B shows the calculated values of log *K* based on reaction iii plotted *versus* the experimental log *K* and in comparison to cases i and ii, *i.e.*, without counterion association and only implicit and explicit solvation. The new calculated values (grey line) are now in the same range as the experimental values (4–14) and the obtained correlation (*R*^2^) between the calculated log *K* and the experimental is improved to *ca.* 0.80 (grey line in Fig. 5B) indicating a rather good agreement between the experimental and theoretical values. Hence, the counterion (Na^+^) association to the ligand anion significantly improves the calculated log *K* values.

Similar to reactions i and ii in reaction iii the Δ*G* of reaction is the difference between summation of Gibbs free energies of all product molecules and summation of Gibbes free energies of all reactant molecules.Δ*G*_iii_ = *G*(Na_(*n*−2)_CaL) + *G*(Na_2_·6H_2_O) − *G*(Na_*n*_L) − *G*(Ca·6H_2_O) = Δ*G*(f,Na_(*n*−2)_CaL) − Δ*G*(f,Na_*n*_L)Δ*G*_iii_ = −*RT* ln(*K*(Na_(*n*−2)_CaL)/*K*(Na_*n*_L))

5 log[*K*(Na_(*n*−2)_CaL)] = log *K*_iii_ + log(*K*(Na_*n*_L))

The values of log(*K*(Na_*n*_L)), which are considerably small due to the very weak binding interaction with the ligands, were reported in previous experimental investigations in literature.^50–55^

For a better comparison, the calculated Δ*G* and log *K* as well as the measured log *K* values are reported in Table S1 in SI. The impact of counterion association was examined by addition of K^+^ cation (instead of Na^+^) and similar results to Na^+^ were obtained using EDA and complexation energies (Tables S13 and S14). Consider that continuous solvent models exhibit a strong dependence of their accuracy on the total charge of the solute. This causes larger ligand-Ca^2+^ electrostatic interactions which yield larger errors and poorer estimations of log *K*. Since the absolute error of solvation energies (or energies in solution) is much larger for ionic species than for neutral species, as a result of the global charge reduction, the electrostatic interaction with the continuum solvent model is also reduced, thus the free energy in solution (or the solvation energy) of the complexes is reduced.^56–61^

### 3.3 Conformational flexibility of the chelator structure

**Fig. 6:**
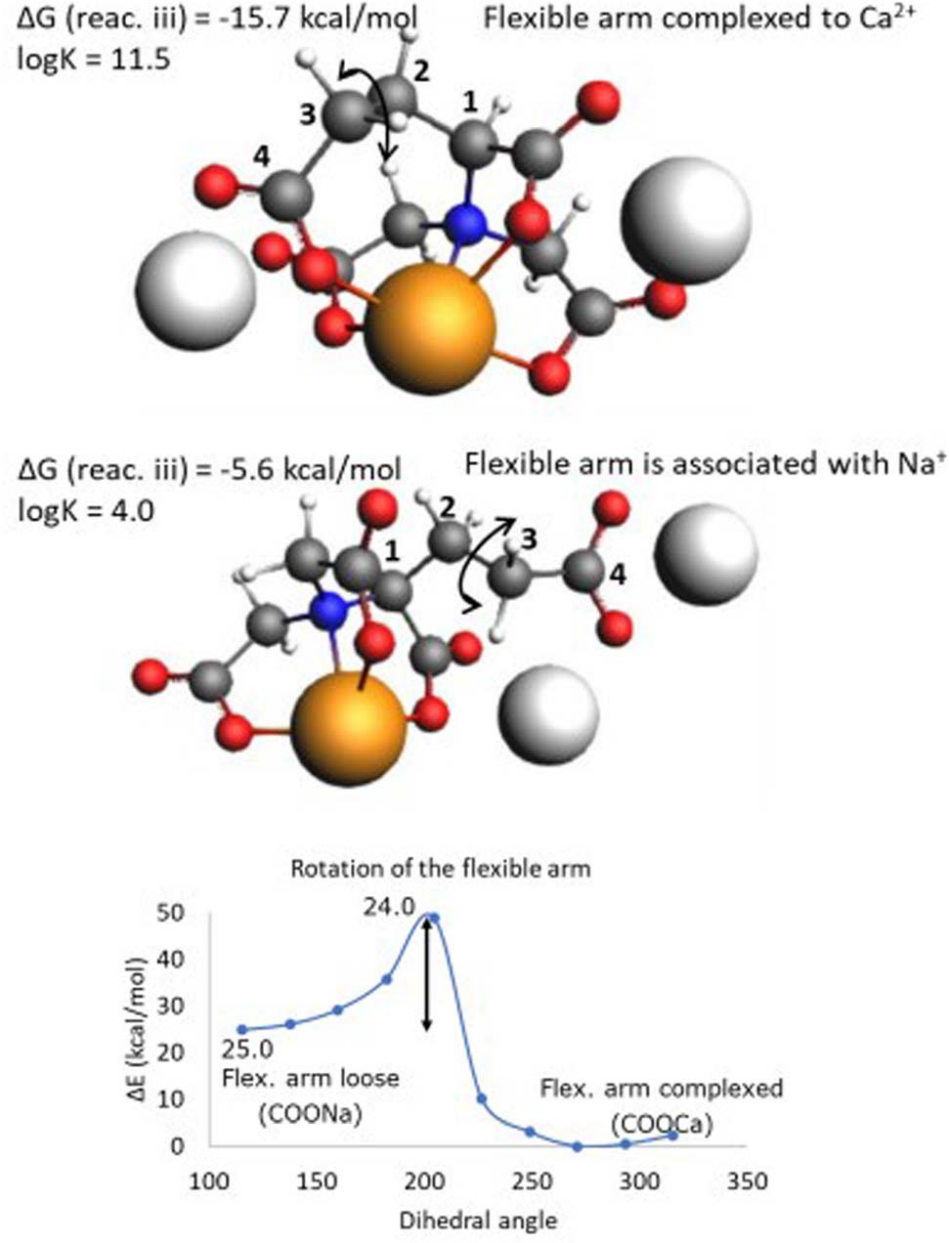
The calculated molecular structures of GLDA chelators in various complexation states, *i.e.*, flexible arm is complexed to Ca^2+^ (top) and associated to Na^+^ (middle). The lower part shows a linear transit scan of the stabilization energy around the dihedral angle.

As can be seen in Fig. 5, GLDA and HIDA deviate from the general trend of the obtained Δ*G* values compared to the rest of the chelators and also the experimental trend, *i.e.*, for GLDA the calculations overestimate the experimental value (11.52 *vs.* 5.9, respectively) and for HIDA underestimate the experimental value (4.9 *vs.* 5.3, respectively). To elucidate this deviating behavior, we analyzed the molecular structures of these two chelators in more detail. As shown in Fig. 6, GLDA has a more flexible arm consisting of two methylene (CH_2_) units, that can be either complexed to the Ca^2+^ cation or freely associated to a Na^+^ counterion in the reaction medium. The arrow shows a selected dihedral (C–C bond) angle that, when rotated, results in either a free (associated with Na^+^) or complexed to Ca^2+^ molecular structure. The calculated Δ*G* values (of reaction iii) depicted next to each structure in Fig. 6 (top and middle) demonstrate a large difference in stabilization energies between the two ways of complexation for the GLDA⋯Ca^2+^ structure, *i.e.*, −15.7 *vs.* −5.6 kcal mol^−1^. A linear transit scan around the highlighted dihedral angle results in a 24 kcal mol^−1^ barrier of rotation starting from the three coordinated molecular complex (flexible arm associated with Na^+^) to the four coordinated complex. This relatively high rotational barrier indicates that the molecular complex with flexible arm associated with Na^+^ can be thermodynamically stable in the solution at room temperature. Previous studies also confirmed that in the higher coordination numbers the strain on the ligand back bone forces one or more coordinating atoms (COO^−^) much farther away.^12^

For a better overview of the flexibility of CH_2_–CH_2_ linkage that causes COO^−^ either associated to Na^+^ or complexed to Ca^2+^ we performed a dihedral scan starting from the initial optimized geometry at each potential well and slightly changed the dihedral angle in 5° steps to sample more local minima around the optimized geometries. It turned out that three coordinated state (flexible arm associated with Na^+^ cation) can include several geometries with very close energies (within 1 kcal mol^−1^) and feasible in solution that can be stabilized through interaction with solvent.^62^ For the four coordinated state (flexible arm complexed to Ca^2+^ cation), the variation of the relative energies of rotation of the dihedral angle are larger and the most stable conformer is at least 1.24 kcal mol^−1^ lower in energy than the others. The results are summarized in Table S3 in the SI. This observation also indicates that the experimentally measured log *K* value can be an average between many cases (states) between the two depicted extremes in Fig. 6 due to the conformational flexibility in solution, and this can affect the calculated Δ*G* correlated to reaction iii. Even at room temperature, multiple association/dissociation equilibria exist in which one or more donor groups may temporarily detach from the metal, rotate, and reattach without complete metal–ligand dissociation (one of the factors underlying the stability of polydentate ligands). Furthermore, to study the effect of temperature, the barrier for rotation around the corresponding dihedral angle (shown in the lowest part of Fig. 6) was calculated at four different temperatures that are relevant to a range of industrial processes where chelators are used to control water hardness ions, *i.e.* 30, 40, 60 and 90 °C (depicted in Fig. S1 in the SI). As example, increasing the temperature from 25 to 90 °C reduces the barrier by 40 percent and increases the chance for a faster transition between three and four coordinated states.

### 3.4 Effect of pH

**Fig. 7:**
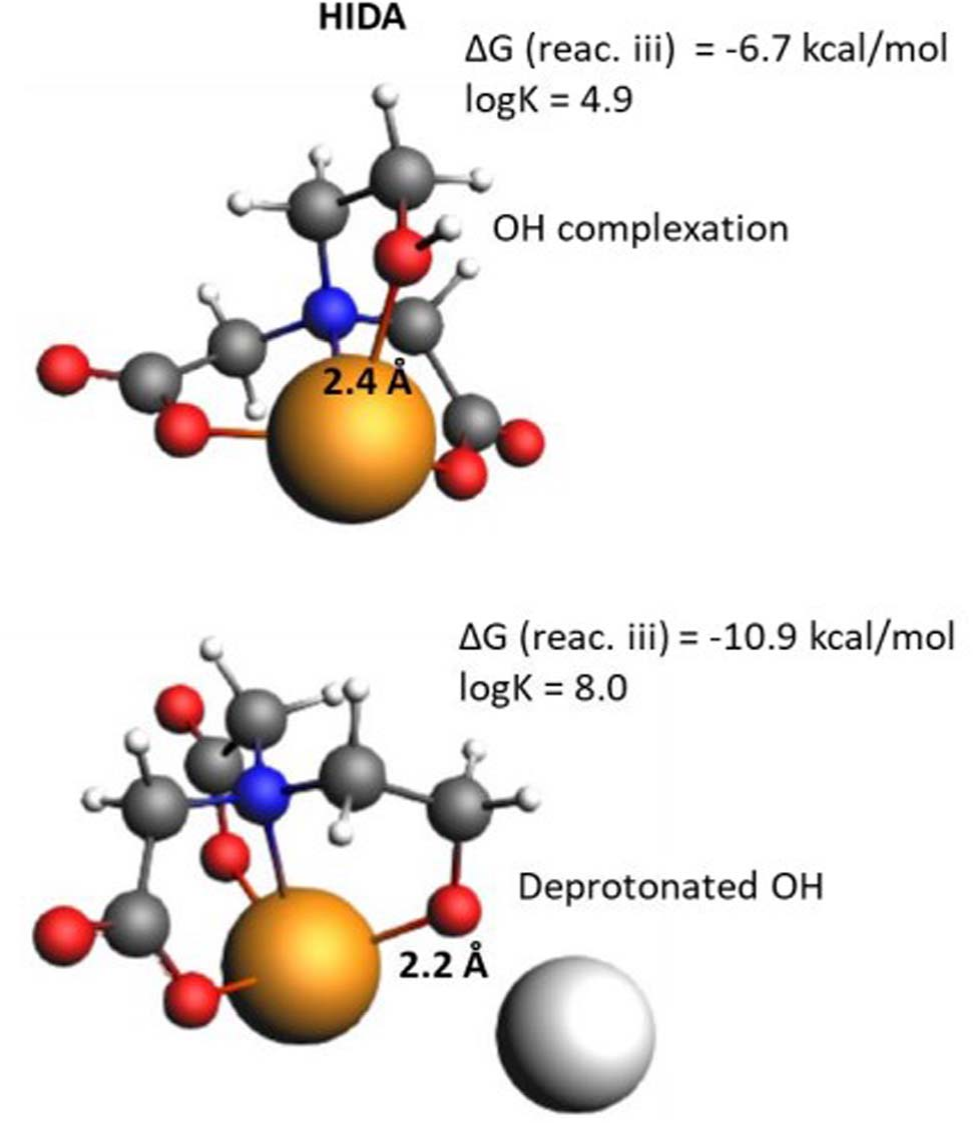
The calculated molecular structures of HIDA chelators in various complexation states, *i.e.*, protonated OH complexed to the Ca^2+^ (up) and deprotonated O^−^ complexed to Ca^2+^ (down).

In the case of HIDA, the molecule contains a CH_2_OH group (*versus* a COO^−^ group in the analogous NTA) that can be deprotonated in basic solution forming an anionic coordination site for complexation to the Ca^2+^ cation. This results in stronger complexation and lower Δ*G* of reaction iii as depicted in Fig. 7. However, at a pH lower than the p*K*_a_ of this OH group, protonation occurs, significantly reducing coordination and hence the log *K* value. Therefore, for molecules like HIDA, there is a very strong pH effect on log *K*, that should be included in the comparison with experimental data.

**Fig. 8:**
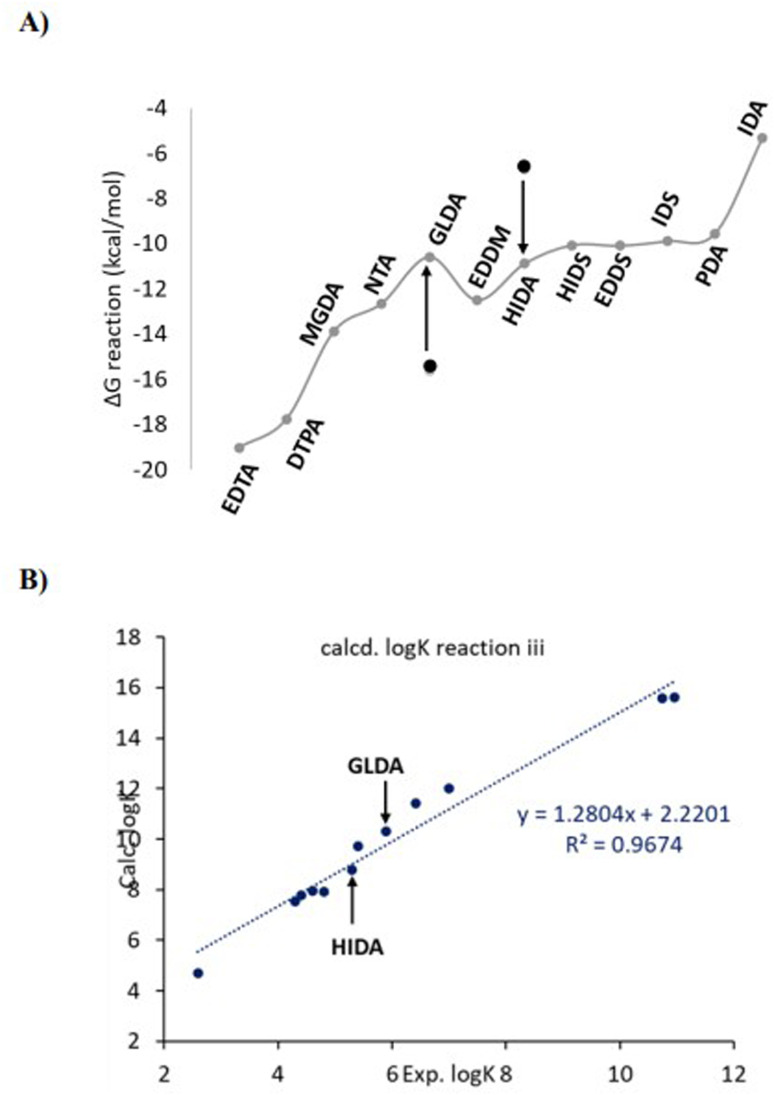
(A) The Δ*G* values of reaction iii, the black dots show the initial values of Δ*G* for HIDA and GLDA before corrections, *i.e.*, GLDA with four coordination to the Ca^2+^ and protonated O–H of HIDA. (B) the log *K* plots including the average between deprotonated (50%) and protonated (50%) complexations for HIDA and the average between three (50%) and four (50%) coordinated states for GLDA.

A significantly improved value for *R*^2^ (0.97) for the log *K* plot can be obtained by including the influence of partial coordination (flexibility) in GLDA, as well as the pH on the coordination of HIDA (Fig. 8).

In a realistic reaction solution with a very dynamic situation in the experimental measurements it is possible that a series of transient states are available in between the two extremes for both GLDA and HIDA, which can be sampled by Boltzmann averaging of the calculated energetic states. We note that addition of an anionic counterion such as HCO_3_^−^ to the reaction iii (Scheme 2) for neutralization of the positive charge of Ca^2+^ was examined. It turned out the *R*^2^ value remains constant, however, the calculated log *K* values for each ligand slightly decreases (0.4 units). The results are shown in Fig. S2 in SI, log *K* and Δ*G* values are reported in Table S2.

**Fig. 9:**
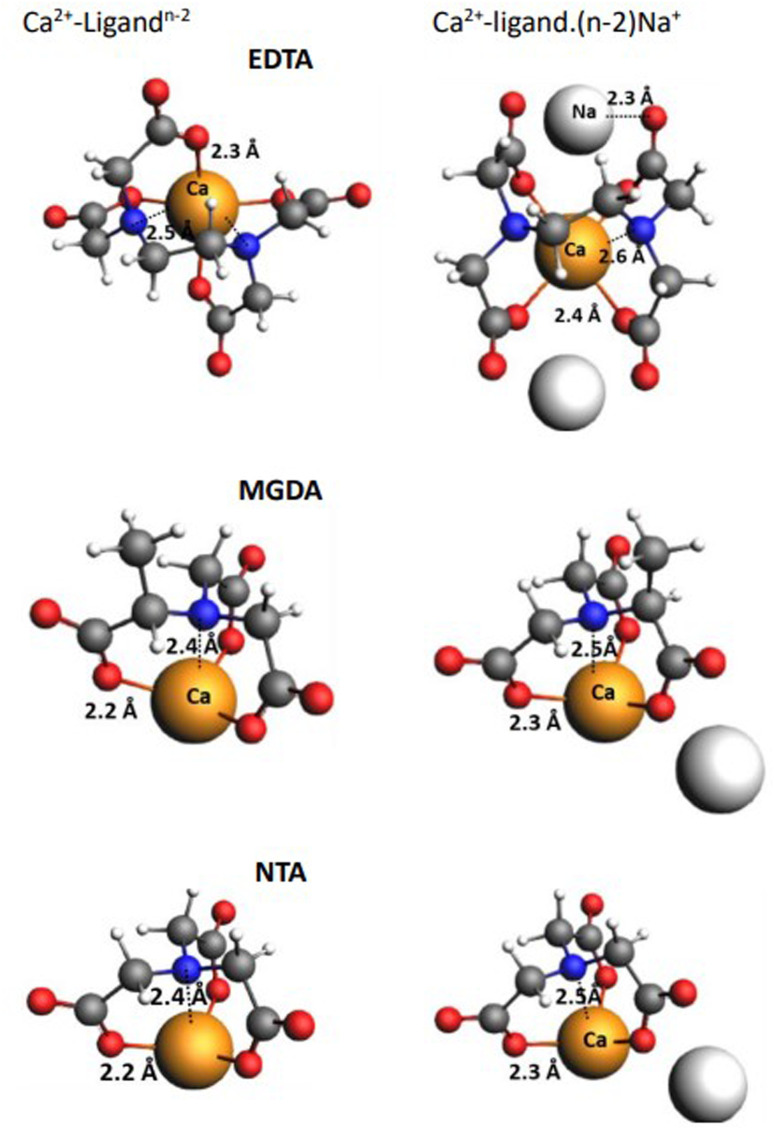
The corresponding structures of EDTA, MGDA, and NTA without Na^+^ association (left) and with Na^+^ association (right), numbers show the distances in Å. According to the crystallographic measurements, the Ca–O and Ca–N bond distances are 2.408 Å and 2.667 Å, respectively in Ca-EDTA crystal.^63^

In Fig. 9, we have depicted both complexation types, *i.e.*, the product complex in reaction iii (Scheme 2) in the presence of Na^+^ and without Na^+^ counterion in reactions Scheme 1. The bond distances between N(tertiary)⋯Ca^2+^ and O(carboxylate)⋯Ca^2+^ for all complexes are shown in the figure. The distances between Ca^2+^ and N/O indicate that all N atoms and carboxylate groups are involved in the complexation. As can be seen in Fig. 9, association of the Na^+^ counterions to the structure of the molecular complexes results in an increase in the Ca^2+^⋯N and Ca^2+^⋯O distances (0.1 Å), which is in line with the weaker complexation (interaction) and less negative Δ*G* of the reaction iii. Moreover, complexation of oxygen to the Ca^2+^ has a shorter bond distance (stronger complexation) than nitrogen, which is correlated to hard acid–base (earth alkaline metals and oxygen, respectively) interaction. According to the energy decomposition analysis (EDA), the dominant interaction between Na^+^-associated ligand and Ca^2+^ are electrostatic interactions.

## 4 Conclusion

We have developed a modified approach for the theoretical calculation of the log *K* values for the Ca^2+^ coordination by aminopolycarboxylates. By including charge stabilization in the chelators *via* Na^+^ cations association, as well as implicit water solvation in the calculations, conformational flexibility and pH effects, the regression coefficient of the calculated values *versus* experimentally determined ones for a series of 12 amino carboxylate chelators increased from 0.55 to 0.97. Previous works explained the importance of isodesmic reactions which yield good results for calculating relative energies using DFT and continuum solvation methods.^23,26^ In this work we highlighted the influence of association of alkali metal cations such as Na^+^ and K^+^ neutralizing the negative charge of molecular complex between central metal atom (Ca^2+^) and multidentate aminocarboxylate ligands. On the one hand, the charge neutralization is an individual physical characteristic that can substantially reduce the electrostatic interaction and hence calculated reaction free energies and log *K* values toward the experimentally measured binding affinities. On the other hand, when the Na^+^ or K^+^ ions are present, the complexation energies of the remaining ligand groups with Ca^2+^ (which are basically coming from the electrostatic term) are numerically much more similar. This means that the absolute errors in the free energies in solution of each species will be also similar (regardless of the counterion's nature), implying that when calculating differences of free energies between reactants and products, better cancellation of errors will be obtained in free energy of reaction with the presence of Na^+^ than in free energy of reactions without Na^+^. A 1 : 1 ratio of metal–ligand complexes that is included in this work, and previous studies may still be a potential source of deviation from experimental values, *i.e.* contribution of the other metal : ligand ratios. The discussed modification in calculation of the log *K* values (considering the counterion association) currently serves as a prediction basis for designing new bio-based chelators for the real application in industry. By analysing the causes of two specific outliers (GLDA and HIDA), we have shown that flexibility of complexation sites of chelators and presence of kinetic barriers for switching between various chelator conformers as well as pH effects can cause significant deviations between calculations and experiments. Full coordination of all available functionalities results in an over-estimation of the calculated log *K*, due to a thermodynamically stronger complexation between ligand and metal cations. Furthermore, there is the possibility of higher M : L stoichiometry such as ML_2_, M_2_L_3_, *etc.* that can still improve the average value of the calculated log *K* with respect to experiment. Understanding the specifics of individual chelator molecules helps in improving the correlations. We are currently expanding the developed methodology to other metal ions, and using modeling to differentiate between predicted log *K* values of not-yet synthesized novel chelators.

## Conflicts of interest

There are no conflicts to declare.

## Supplementary Material

RA-016-D5RA07375H-s001

## Data Availability

The authors declare that the data supporting this article have been included as part of the supplementary information (SI). Supplementary information: includes all *xyz* coordinates of ligand–Na and ligands–Na–Ca, Δ*G* and log *K* for the reactions i, ii and iii, bond distances for ligand–Ca and ligand–Ca–Na, tables with calculated Δ*G*, relative Δ*G* and Boltzmann weights of the distribution of molecular structures at three and four Ca^2+^ coordinated states, plot of variation of the activation barriers *vs.* temperature for the transition between three-coordinated to four coordinated system, EDA results, conformational search, details of thermodynamic calculation of the log *K* values. See DOI: https://doi.org/10.1039/d5ra07375h.
