# Supplementary material for: The importance of counterion association in the calculated binding constants of Ca2+-aminopolycarboxylate complexes
Source: RSC Adv. 2026 Mar 18;16(17):15165–75. doi: 10.1039/d5ra07375h (PMC12997534; doi:10.1039/d5ra07375h)
Supplement: RA-016-D5RA07375H-s001 [file RA-016-D5RA07375H-s001.pdf]

Supplementary Information (SI) for RSC Advances.
This journal is © The Royal Society of Chemistry 2026

Supplementary Information for:

Signature of counterion association in the calculated binding constants of Ca^{2+}-aminopolycarboxylate complexes.

Mojgan Heshmat,[a*] Pavlo Kostetskyy[b], Guanna Li[c] , Daan S. van Es[a*]

[a]Wageningen Food & Biobased Research, Bornse Weilanden 9, 6708 WG Wageningen, The Netherlands

[b]Archer Daniels Midland Company, 1001 N Brush College Road, Decatur, IL, 62521, USA

[c]Biobased Chemistry & Technology, Wageningen University, Bornse Weilanden 9, 6708 WG, Wageningen, The Netherlands.

In equation 1 below, ΔG(prd.) is summation of the Gibbs free energy of all compounds on the product side, i.e., for method ii: G(Ca^{2+}.ligand^{n-}) + 12G(H_2O) and ΔG(react.) is summation of Gibbs free energy of all reactant species i.e., G(Ca^{2+}.6H_2O) + G(ligand^{n-}). This applies for other reaction equations related to method i and method iii. Hence, for reaction equation i: G(Ca^{2+}.chelate^{n-}) + 6G(H_2O) – G(Chelate^{n-}) – G(Ca^{2+}.6H_2O) and the same for reaction equation iii: G((n-2)Na^{+}.Ca^{2+}.chelate^{n-}) + G(2Na^{+}.6H_2O) – G(Ca^{2+}.6H_2O) - G(Chelate^{n-}.nNa^{+}). We included this in the text (in blue right after equation 1 and 2) and with more detailed in the SI.

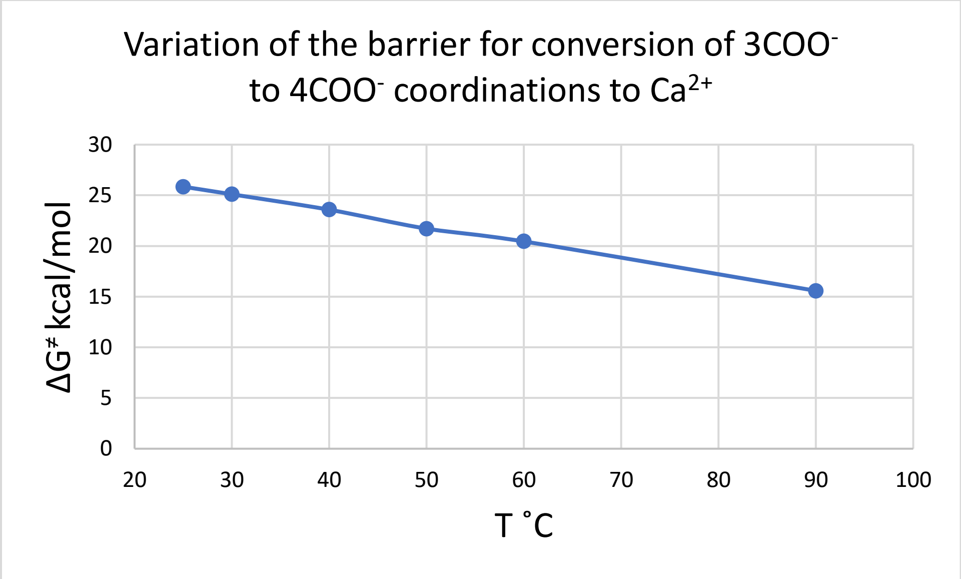

Figure S1. Variation of the barrier against T for the conversion from 3COO^- to 4COO^- coordination to Ca^{2+}.

Table S1. The calculated ΔG (kcal/mol) and logK values of reaction **of method ii** and **iii** in the main text.

Chelator	ΔG complexation Ca^{2+}···Chelator	ΔG reaction ii	logK reaction ii	ΔG reaction iii	logK reaction iii	Exp. logK
EDTA	-125.19	-66.7641175	50.62	-19.04	14.01	10.96
DTPA	-140.79	-76.14838	56.06	-17.77	13.08	10.74
MGDA	-93.20	-41.5411275	30.58	-13.88	10.22	7.0
NTA	-91.07	-40.911745	30.12	-12.65	9.31	6.41
GLDA	-106.49	-45.8445225	35.96	-10.6(-15.65)	7.80(11.51)	5.9
EDDM	-113.48	-51.3113025	37.77	-12.51	9.21	5.4
HIDA	-77.716	-14.61071	10.75	-10.89(-6.70)	8.02(4.93)	5.3
HIDS	-99.48	-38.6483525	26.24	-10.07	7.42	4.8
EDDS	-113.98	-72.941855	51.63	-10.10	7.44	4.6
IDS	-101.47	-39.50238	29.08	-9.90	7.29	4.4
PDA	-64.21	-11.82963	10.50	-9.56	7.03	4.3
IDA	-62.03	-14.26684	8.71	-5.31	3.91	2.59

$$Ca^{2+}(HCO_3)^-_2.4H_2O + Chelator^{n-}.nNa^+ \longrightarrow (n\text{-}2)Na^+.Ca^{2+}.chelator^{n-} + 2Na^+(HCO_3)^-.4H_2O \quad \textbf{(2)}$$

$$(n = 2 - 5)$$

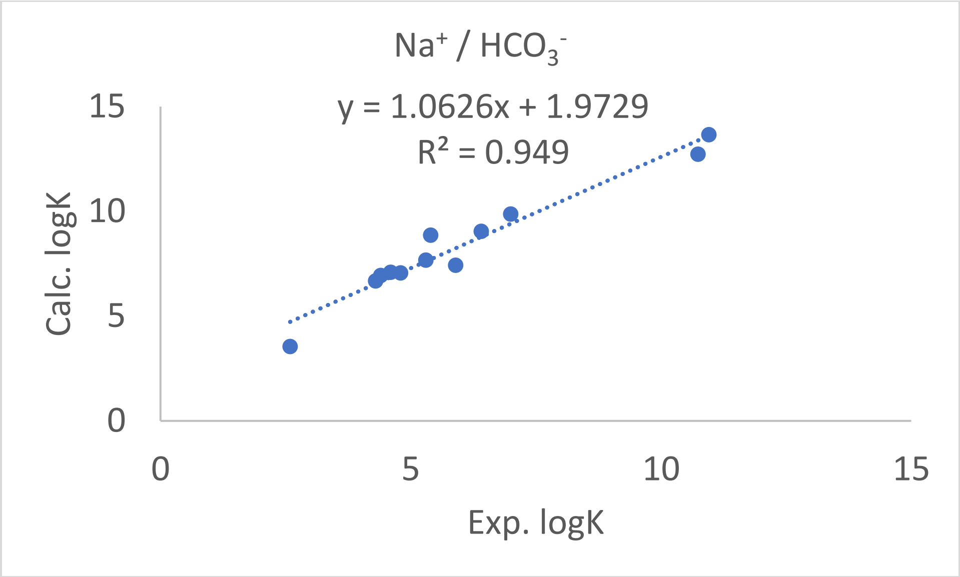

Figure S2. The correlation between calculated and experimental logK values using Na^+ and HCO_3^- counterions for $chelator^{n-}$ and Ca^{2+} neutralization, respectively (reaction **iii**, above).

Table S2. The calculated ΔG (kcal/mol) and logK values of reaction **iii** corresponding to Figure S2 (including HCO_3^- as the counterion for Ca^{2+})

Chelator	ΔG reaction iii	logK reaction iii	Exp. logK
EDTA	-18.56	13.66	10.96
DTPA	-17.29	12.73	10.74
MGDA	-13.40	9.87	7.0
NTA	-12.29	9.05	6.41
GLDA	-10.09	7.42	5.9
EDDM	-12.03	8.85	5.4
HIDA	-10.41	7.67	5.3
HIDS	-9.59	7.07	4.8
EDDS	-9.62	7.08	4.6
IDS	-9.42	6.94	4.4
PDA	-9.08	6.68	4.3
IDA	-4.83	3.55	2.59

Table S3. Variation of the dihedral angle shown in **Figure 6** in the main text, in small steps of 5° to test the stability of the three coordinated molecular complex at its local minimum structure.

**Four COO^- complexed to Ca^{2+}**			**Three COO^- complexed to Ca^{2+}**		
Dihedral	ΔG	BW	Dihedral angle	ΔG	BW
245	8.09	0.01	125	0.82	0.09
250	7.35	0.01	130	0.08	0.12
255	6.20	0.02	135	0.01	0.12
260	5.19	0.03	140	0.04	0.12
265	4.39	0.04	145	0.26	0.11
270	3.62	0.05	150	0	0.12
275	2.26	0.09	155	0.53	0.10
280	1.56	0.12	160	1.23	0.08
285	1.24	0.13	165	2.17	0.05
290	0	0.23	170	4.91	0.02
295	1.48	0.12	175	3.59	0.03
300	2.32	0.09	180	5.00	0.02
305	3.30	0.06	185	4.64	0.02

Table S4. The calculated bond distances (in Å) related to the molecular complex between ligands and Ca^{2+} cation in the presence and without the Na^{+} counterions.

Distance Å	$N^{1}\cdots Ca^{2+}$	$N^{2}\cdots Ca^{2+}$	$O^{1}\cdots Ca^{2+}$	$O^{2}\cdots Ca^{2+}$	$O^{3}\cdots Ca^{2+}$	$O^{4}\cdots Ca^{2+}$
$EDTA^{4-}$	2.55	2.55	2.33	2.32	2.33	2.32
$[EDTA\text{-}2Na^{+}]^{2-}$	2.57	2.57	2.37	2.37	2.37	2.37
$DTPA^{5-}$	2.66	2.61	2.23	2.31	2.37	2.37
$[DTPA\text{-}3Na^{+}]^{2-}$	2.72	2.71	2.42	2.44	2.45	2.36
$MGDA^{3-}$	2.42		2.20	2.20	2.20	
$[MGDA\text{-}1Na^{+}]^{2-}$	2.54		2.31	2.28	2.28	
$[NTA]^{3-}$	2.42		2.20	2.20	2.20	
$[NTA\text{-}1Na^{+}]^{2-}$	2.53		2.32	2.28	2.28	
$[GLDA]^{4-}$	2.40		2.25	2.23	2.22	
[a]$[GLDA\text{-}2Na^{+}]^{2-}$(4)	2.58		2.32	2.32	2.37	2.36
[b]$[GLDA\text{-}2Na^{+}]^{2-}$(3)	2.53		2.29	2.27	2.33	
$[EDDM]^{4-}$	2.51	2.51	2.33	2.33	2.34	2.34
$[EDDM\text{-}2Na^{+}]^{2-}$	2.53	2.53	2.38	2.38	2.38	2.38
[c]$[HIDA\text{-}OH]^{2-}$	2.54		2.27	2.27	2.42(OH)	
[d]$[HIDA\text{-}O^{-}Na^{+}]^{2-}$	2.52		2.30	2.32	2.23(O^{-})	
$[HIDS]^{4-}$	2.33		2.37	2.30	2.28	2.28
$[HIDS\text{-}2Na^{+}]^{2-}$	2.44		2.38	2.42	2.42	2.31
$[EDDS]^{4-}$	2.51	2.51	2.33	2.33	2.31	2.31
$[EDDS\text{-}2Na^{+}]^{2-}$	2.53	2.53	2.39	2.39	2.30	2.30
$[IDS]^{4-}$	2.33		2.25	2.31	2.29	2.38
$[IDS\text{-}2Na^{+}]^{2-}$	2.38		2.15	2.48	2.38	2.42
$[IDA]^{2-}$	2.50		2.28	2.28		
$[PDA]^{2-}$	2.41		2.33	2.33		

Table S5. Comparison of the complexation energies (in kcal/mol) between Ca^{2+} and ligands anion with fully negative charge and neutralized ligands with sodium counterion: 1) no counterion association 2) with counterion association

Ligand with anionic charge > -2	No counterion association - Complexation energy $Ca^{2+}.ligand^{n-}$	With counterion association- Complexation energy $Ca^{2+}.(n-2)Na^{+}.ligand^{n-}$
EDTA	-125.19	-72.08
DTPA	-140.79	-73.08
MGDA	-93.20	-85.83
NTA	-91.07	-83.02
GLDA (four coord.)	-106.49	-53.99
EDDM	-113.48	-97.48
HIDS	-99.48	-79.75
EDDS	-113.98	-70.08
IDS	-101.47	-80.09

Energy Decomposition Analysis (EDA)

In the Energy Decomposition Analysis (EDA) approach, the total interaction energy between the two fragments at any specific molecular structure is divided into its various components including ΔE_{Pauli} that is the interaction between the occupied molecular orbitals and is responsible for the steric repulsion, ΔE_{elstat} that is the classical electrostatic interaction between the two fragments. ΔE_{steric} is the sum of ΔE_{Pauli} and ΔE_{elstat}. ΔE_{oi} is the orbital interaction that accounts for the charge transfer between the HOMO and LUMO of the two fragments (covalent interaction); finally, ΔE_{disp} accounts for the dispersion energy due to the van der Waals and long range attractions. ΔE_{int} is the sum of the electrostatic, Pauli, and orbital interactions plus dispersion contribution. We calculate EDA between Ca^{2+} and the rest of molecular complex.

$$\Delta E_{int} = \Delta E_{Pauli} + \Delta E_{elstat} + \Delta E_{oi} + \Delta E_{disp}$$

Table S6: EDA results (in kcal/mol) for neutralized ligands with Na^+ counterion: the interaction energies are calculated between Ca^{2+} and the rest of molecular complex (Na^+(n-2).Ligand)

Ligand with anionic charge > -2	Pauli	**Elstat**	Steric	Orbital interaction	Disp.	Total
EDTA	99.27	**-510.41**	-411.14	-170.90	-17.13	-599.18
DTPA	98.41	**-523.37**	-424.96	-195.02	-23.11	-643.08
MGDA	92.58	**-514.97**	-422.39	-151.54	-9.84	-583.77
NTA	91.75	**-514.50**	-422.75	-150.27	-9.63	-582.66
GLDA (four coord.)	89.11	**-507.09**	-417.98	-161.77	-13.06	-592.82
EDDM	96.90	**-536.21**	-439.30	-175.74	-14.10	-629.14
HIDS	84.84	**-501.44**	-416.60	-171.90	-12.09	-600.59
EDDS	100.36	**-510.38**	-410.02	-201.24	-15.79	-627.05
IDS	110.86	**-515.80**	-404.94	-179.31	-13.18	-597.44

Table S7: EDA results (in kcal/mol) for anionic ligands (not neutralized): the interaction energies are calculated between Ca^{2+} and the rest of molecular complex ($ligand^{n-}$)

Ligand with anionic charge > -2	Pauli	**Elstat**	Steric	Orbital interaction	Disp.	Total
EDTA	110.51	**-826.23**	-715.72	-176.36	-14.10	-906.17
DTPA	99.26	**-896.77**	-797.51	-181.40	-15.86	-994.77
MGDA	132.38	**-701.07**	-568.69	-162.75	-8.72	-740.17
NTA	131.55	**-702.46**	-570.92	-160.87	-8.60	-740.38

GLDA (four coord.)	124.31	**-798.29**	-673.98	-169.08	-10.98	-854.03
EDDM	111.63	**-849.04**	-737.41	-169.23	-11.79	-918.43
HIDS	118.31	**-840.43**	-722.12	-170.79	-10.01	-902.92
EDDS	107.67	**-830.39**	-722.72	-176.38	-13.37	-912.47
IDS	119.77	**-846.17**	-726.40	-170.77	-10.25	-907.42

Conclusion based on Tables S5 – S7, neutralization is basically an inherent physical characteristic of counterion association.

The effect of water concentration

$K_{exp} = [CaL]/([Ca\cdot 6H_2O][L\cdot 6H_2O])$

$K_{ii} = ([CaL][H_2O]^{\wedge}12)/([Ca\cdot 6H_2O][L\cdot 6H_2O])$

$K_{ii} = K_{exp} \cdot [H_2O]^{\wedge}12$

Therefore, to calculate K_{exp} from ΔG_{ii} one should calculate:

$K_{ii} = \exp(-\Delta G_{ii}/RT)$

And then, correct K_{ii} to obtain K_{exp}:

$K_{exp} = K_{ii} \cdot [H_2O]^{\wedge}(-12)$

Or directly from ΔG_{ii}:

$K_{exp} = \exp(-\Delta G_{ii}/RT) \cdot [H_2O]^{\wedge}(-12)$

In the logarithmic form:

$\log(K_{exp}) = -(\Delta G_{ii},1M)/RT\ln 10 - 12 \log[H_2O]$

In summary, first ΔG_{ii} is calculated by including all the present water molecules. Second, to obtain $\log(K_{exp})$, the term $12\log[H_2O]$ has to be considered to correct K_{ii}.

Similarly, for reaction i one should calculate ΔGi by taking into account the 6 water molecules on the products side and later subtract the term $6\log[H_2O]$ to obtain log(Kexp):

log(Kexp) = -(ΔGi,1M)/RTln10 - 6 log[H2O]

Table S8: Reaction i with removing 6 $\log[H_2O]$ in order to obtain a closer correlation with respect to the experimental logK values.

Ligands	logK(exp)	logK(reaction i,calc.) 1 atm -> 1M	log(K_calcd) = logK(reaction i,calc.) -6 $\log[H_2O]$
EDTA	10.96	63.21933054	52.77933054
DTPA	10.74	74.70180723	64.26180723
MGDA	7.0	39.66814998	29.22814998
NTA	6.41	38.10210572	27.66210572
GLDA	5.9	51.66149732	41.22149732
EDDM	5.4	54.59638599	44.15638599
HIDA	5.3	28.26697824	17.82697824
HIDS	4.8	44.29444888	33.85444888
EDDS	4.6	54.96733983	44.52733983
IDS	4.4	45.76140181	35.32140181
PDA	4.3	18.32329016	7.88329016
IDA	2.59	16.72306087	6.28306087

Table S9: Reaction ii with removing 12 $\log[H_2O]$ in order to obtain a closer correlation with respect to the experimental logK values.

Ligands	logK(exp)	logK(reaction ii,calc.) 1 atm -> 1M	log(K_calcd) = logK(reaction ii,calc.) -12 $\log[H_2O]$
EDTA	10.96	33.84573919	12.94573919
DTPA	10.74	40.75435035	19.85435035

MGDA	7.0	15.27679676	-5.62320324
NTA	6.41	14.81345092	-6.08654908
GLDA	5.9	18.44491815	-2.45508185
EDDM	5.4	22.46951329	1.56951329
HIDA	5.3	-4.549138713	-25.44913871
HIDS	4.8	13.14716136	-7.75283864
EDDS	4.6	38.39373497	17.49373497
IDS	4.4	13.77588886	-7.12411114
PDA	4.3	-6.596545243	-27.49654524
IDA	2.59	-4.80229277	-25.70229277

Calculation of the conversion factor of ΔG 1atm→1M

Free energies calculated with Gaussian using ideal-gas partition functions are referenced to 1 atm, whereas the experimentally determined K in solution is referenced to a concentration of 1 M. The free energy of standard-state change must be considered. This term has no effect when the number of moles of reactants and products is the same, but this is not the case in equation 1. For each species, there is a term

ΔG(1atm→1M) = G(1M) - G(1atm) = -RTln(C_1M/C_1atm) =

= -RT ln(1molL^(-1))/((1atm/(0.0820atmLK^(-1) mol^(-1) 298K))) ≅ **1.89kcal·mol^(-1)**

Thus, the free energy of reaction 1 for a 1 M standard state is:

ΔG1,1M = 12[G(H2O,1atm)+**ΔG(1atm→1M)**] + [G(CaL,1atm)+**ΔG(1atm→1M)**] - [G(Ca·6H2O,1atm)+**ΔG(1atm→1M)**] - [G(L·6H2O,1atm)+**ΔG(1atm→1M)**] = ΔG1,1atm+11**ΔG(1atm→1M)**

Addition of a constant term does not change the trade-off and hence the R^2 value.

Table S10. Addition of 1atm to 1M correction results in smaller logK values than previously calculated with the same R^2 but different intercept.

Ligand (Energies in ha)	G(Ca.Ligand)	G(Ligand.6H2O)	ΔG reaction (ha)	ΔG reaction ii (kcal/mol)	ΔG reaction ii (1M); added 11ΔG(1atm→1 M)	Log K (ii) (1M)
EDTA	-1777.970728	-1559.065301	-0.106397	-66.7641175	-45.9741175	33.84573919
DTPA	-2139.394568	-1920.474186	-0.121352	-76.14838	-55.35838	40.75435035
MGDA	-1455.830334	-1236.965103	-0.066201	-41.5411275	-20.7511275	15.27679676
NTA	-1416.525139	-1197.660911	-0.065198	-40.911745	-20.121745	14.81345092
GLDA	-1683.302465	-1464.430376	-0.073059	-45.8445225	-25.0545225	18.44491815
EDDM	-1699.370867	-1480.490066	-0.081771	-51.3113025	-30.5213025	22.46951329
HIDA	-1342.886513	-1124.064199	-0.023284	-14.61071	6.17929	-4.549138713
HIDS	-1719.248956	-1500.388335	-0.061591	-38.6483525	-17.8583525	13.14716136
EDDS	-1777.980573	-1559.065301	-0.116242	-72.941855	-52.151855	38.39373497
IDS	-1644.005468	-1425.143486	-0.062952	-39.50238	-18.71238	13.77588886
IDA	-1189.035561	-970.217679	-0.018852	-11.82963	8.96037	-6.596545243
PDA	-1302.204625	-1083.382859	-0.022736	-14.26684	6.52316	-4.80229277

G H2O	-76.452265
G Ca(H2O)6	-1136.22621

Table S11. Addition of 1atm to 1M correction results in smaller logK values than previously calculated with the same R^2 but different intercept.

Ligand (Energies in ha)	G(Ca.Ligand)	G(Ligand)	ΔG reaction i (kcal/mol)	ΔG reaction i (1M); added 5ΔG(1atm→1M)	Log K (i) (1M)
EDTA	-1777.970728	-1100.306198	-95.323525	-85.873525	63.21933054
DTPA	-2139.394568	-1461.705182	-110.920665	-101.470665	74.70180723
MGDA	-1455.830334	-778.216785	-63.3329475	-53.8829475	39.66814998
NTA	-1416.525139	-738.91498	-61.2057225	-51.7557225	38.10210572
GLDA	-1683.302465	-1005.667735	-79.624025	-70.174025	51.66149732
EDDM	-1699.370867	-1021.725003	-83.61061	-74.16061	54.59638599
HIDA	-1342.886513	-665.297644	-47.8462475	-38.3962475	28.26697824
HIDS	-1719.248956	-1041.631767	-69.6170475	-60.1670475	44.29444888
EDDS	-1777.980573	-1100.333906	-84.1144925	-74.6644925	54.96733983
IDS	-1644.005468	-966.378729	-71.6096725	-62.1596725	45.76140181
IDA	-1189.035561	-511.471681	-34.33931	-24.88931	18.32329016
PDA	-1302.204625	-624.637281	-32.16565	-22.71565	16.72306087

Reaction iii and associated sodium-ligand concentration

Reaction iii is a metal-exchange reaction mediated by the ligand. Therefore, the calculated free energy is not directly related to the logK of the complex $Na_{n-2}CaL$, but rather to the free-energy difference between the complexes $Na_{n-2}CaL$ and Na_nL. Then, there are two reactants and two products with no release of water molecules. The free-energy difference at 1 atm and 1 M would be the same, and thus:

$\Delta G_{iii} = \mathbf{G(Na_{(n-2)}CaL) + G(2Na\cdot 6H_2O) - G(Na_nL) - G(Ca\cdot 6H_2O)} = \Delta G(f,Na_{(n-2)}CaL) - \Delta G(f,Na_nL)$

$\Delta G_{iii} = -RTln(K(Na_{(n-2)}CaL) / K(Na_nL))$

$log[K(Na_{(n-2)}CaL)] = -(\Delta G_{iii})/(RTln10) + log(K(Na_nL))$

The values of log(K(NanL)), which are considerably small due to the very weak binding interaction with the ligands, were reported in previous experimental investigations in literature.[50-57]

Table S12. The experimental logK values for Na_nL ionic species

Ligand	$logK_{iii}$	Exp. $logK(Na_nL)$	$log[K(Na_{(n-2)}CaL)]$
EDTA	14.01	1.6	15.61
DTPA	13.08	2.5	15.58
MGDA	10.22	1.8	12.02
NTA	9.31	2.1	11.41
GLDA	7.80	2.5	10.30
EDDM*	9.21	0.5	9.71
HIDA	8.02	0.8	8.82
HIDS*	7.42	0.5	7.92
EDDS*	7.44	0.5	7.94
IDS*	7.29	0.5	7.79
PDA*	7.03	0.5	7.53
IDA	3.91	0.8	4.71

* Due to a very weak complexation between these ligands and Na^+ the measured logK values are reported between 0.1 – 0.5 (similar to interaction between alkali metals and amino acids or carboxylate ligands).

Table S12-a. Calculation of $logK(Na_nL)$ using reaction ii including hydrated ligands with six water molecules; $n(Na^+.6H_2O) + L.6H_2O \rightarrow Na_nL + (6n+6)H_2O$; n = negative charge of the ligand (-2 to -5).

Ligands	L.6H_2O (ha)	Na_nL (ha)	ΔG reaction (kcal/mol)	Correction factor for 1atm to 1M (kcal/mol) Reaction ii / must be added to ΔG	Correction factor for nlog[H_2O] Reaction ii must be subtracted from logK	logK(NanL) (including 1 atm -> 1M & nlog[H2O] corrections)
EDTA	-1559.065301	-1749.510426	-51.2623575	49.14 (1.89*26)	52.2(30*1.74)	-50.63753916
DTPA	-1920.474186	-2273.223044	-72.8998125	58.59(1.89*31)	62.64(36*1.74)	-52.10524244
MGDA	-1236.965103	-1265.091451	-20.1847925	39.69(1.89*21)	41.76(24*1.74)	-56.11956146
NTA	-1197.660911	-1225.787505	-7.664285	39.69	41.76	-65.33704848
GLDA	-1464.430376	-1654.850040	-35.28558	49.14	52.2	-62.39950162
EDDM	-1480.490066	-1670.911925	-36.6629425	49.14	52.2	-61.38549952
HIDA	-1124.064199	-989.884880(-1151.677092)	2.6662475	30.24(1.89*16)	31.32	-55.54528873
HIDS	-1500.388335	-1690.803433	-32.420415	49.14	52.2	-64.50881078
EDDS	-1559.065301	-1749.531544	-64.5139025	49.14	52.2	-40.88186878
IDS	-1425.143486	-1615.561451	-34.2194575	49.14	52.2	-63.18437159
PDA	-970.217679	-836.036143	4.057415	30.24	31.32	-56.56945396
IDA	-1083.382859	-949.198434	5.8702625	30.24	31.32	-57.90405627
Na.6H_2O	-620.982823					
H_2O	-76.452265					

Table S12-b. Calculation of logK(Na_nL) using reaction i including free anionic ligands; n(Na^+.6H_2O) + L(n-) → Na_nL + 6nH_2O

Ligands	L(n-) (ha)	Na_nL (ha)	ΔG reaction (kcal/mol)	Correction factor for 1atm to 1M (1.89 kcal/mol) Reaction i must be added to ΔG	Correction factor for 6nlog[H_2O] (1.74) Reaction i must be subtracted from logK	logK(NanL) (including 1 atm -> 1M & 6nlog[H2O] corrections)
EDTA	-1100.306198	-1749.510426	-79.83306	37.8 (20*1.89)	41.76 (24*1.74)	-10.81563253
DTPA	-1461.705182	-2273.223044	-107.6833925	47.25 (25*1.89)	52.2 (30*1.74)	-7.709470279
MGDA	-778.216785	-1265.091451	-41.9879075	28.35 (15*1.89)	31.32 (18*1.74)	-21.27989297
NTA	-738.91498	-1225.787505	-27.9695575	28.35	31.32	-31.60007841
GLDA	-1005.667735	-1654.850040	-66.0763775	37.8	41.76	-20.94318066
EDDM	-1021.725003	-1670.911925	-68.973545	37.8	41.76	-18.81031154

HIDA	-665.297644	-989.884880	-30.580585	28.35 (10*1.89)	20.88 (12*1.74)	-12.2808565
HIDS	-1041.631767	-1690.803433	-59.400405	37.8	41.76	-25.85797265
EDDS	-1100.333906	-1749.531544	-75.697835	37.8	41.76	-13.85994864
IDS	-966.378729	-1615.561451	-66.338045	37.8	41.76	-20.75054336
PDA	-511.471681	-836.036143	-16.2899	28.35	20.88	-22.80153256
IDA	-624.637281	-949.198434	-14.2135025	28.35	20.88	-24.33015806
Na.6H_2O	-620.982823					
H_2O	-76.452265					

Table S12-c. Calculation of logK(Na_nL) using reaction ii including same number of H2O as the COO^-;
$n(Na^+.6H_2O) + L.nH_2O \rightarrow Na_nL + 7nH_2O$; n = negative charge of the ligand (number of COO^-)

Ligands	$L.nH_2O$ (ha) n = COO^-	Na_nL (ha)	ΔG reaction (kcal/mol)	Correction factor for 1atm to 1M (1.89 kcal/mol) Reaction i must be added to ΔG	Correction factor for nlog[H_2O] (1.74) Reaction i must be subtracted from logK	logK(NanL) (including 1 atm -> 1M & nlog[H2O] corrections)
EDTA	-1406.141223	-1749.510426	-63.5324925	45.36 (24*1.89)	48.72 (28*1.74)	-35.34157155
DTPA	-1844.01179	-2273.223044	-79.2588975	56.70 (30*1.89)	60.9 (35*1.74)	-44.29233893
MGDA	-1007.594004	-1265.091451	-29.1662	34.01 (18*1.89)	36.54 (21*1.74)	-40.11332468
NTA	-968.290247	-1225.787505	-16.37273	34.01	36.54	-49.53176429
GLDA	-1311.50424	-1654.850040	-48.84711	45.36	48.72	-46.15282042
EDDM	-1327.567232	-1670.911925	-48.1524675	45.36	48.72	-46.66421009
HIDA	-894.69254	-989.884880	-6.66656	22.68 (12*1.89)	24.36 (14*1.74)	-36.14895307
HIDS	-1347.466375	-1690.803433	-43.361505	45.36	48.72	-50.19127437
EDDS	-1406.172229	-1749.531544	-57.3277725	45.36	48.72	-39.9094316
IDS	-1272.221386	-1615.561451	-45.2483975	45.36	48.72	-48.80216077
PDA	-664.387198	-836.036143	-9.3917925	22.68	24.36	-34.98065634
IDA	-777.551303	-949.198434	-8.2535075	22.68	24.36	-34.14266098
Na.6H_2O	-620.982823					
H_2O	-76.452265					

Table S13. Comparison of the complexation energies (in kcal/mol) between Ca^{2+} and ligands anion with fully negative charge and neutralized ligands with potassium counterion: 1) no counterion association 2) with counterion association

Ligand with anionic charge > -2	No counterion association - Complexation energy $Ca^{2+}.ligand^{n-}$	With counterion association- Complexation energy $Ca^{2+}.(n-2)K^+.ligand^{n-}$
EDTA	-125.19	-81.25
DTPA	-140.79	-86.53
MGDA	-93.20	-86.53
NTA	-91.07	-83.67
GLDA	-106.49	-91.91
EDDM	-113.48	-93.66

HIDS	-99.48	-64.99
EDDS	-113.98	-73.76
IDS	-101.47	-84.98

Table 14. EDA results (in kcal/mol) for neutralized ligands with K^+ counterion: the interaction energies are calculated between Ca^{2+} and the rest of molecular complex (K^+(n-2).Ligand)

Ligand with anionic charge > -2	Pauli	**Elstat**	Steric	Orbital interaction	Disp.	Total
EDTA	101.24	**-521.60**	-420.36	-174.90	-16.83	-612.09
DTPA	101.13	**-533.39**	-432.26	-200.22	-23.53	-656.01
MGDA	93.21	**-518.09**	-424.88	-155.51	-9.92	-590.31
NTA	92.32	**-516.47**	-424.16	-156.05	-9.70	-589.90
GLDA (four coord.)	91.45	**-516.18**	-424.73	-165.58	-14.28	-604.59
EDDM	98.08	**-518.80**	-420.72	-202.72	-14.16	-637.60
HIDS	86.55	**-482.78**	-396.23	-186.44	-13.08	-595.75
EDDS	102.13	**-508.86**	-406.73	-211.93	-16.03	-634.69
IDS	88.06	**-499.10**	-411.03	-182.91	-13.22	-607.17

Table S15. Inclusion of water molecules equal to the number of COO- functional

Ligand (Energies in ha)	G(Ca.Ligand)	G(Ligand.nH2O) n = number of COO^-	ΔG reaction ii (with n H2O) in kcal/mol	logK
EDTA	-1777.970728	-1406.141223	-79.0304875	58.18154678
DTPA	-2139.394568	-1844.01179	-82.5055825	60.73988102
MGDA	-1455.830334	-1007.594004	-50.5168875	37.19008634
NTA	-1416.525139	-968.290247	-49.6145425	36.52578792
GLDA	-1683.302465	-1311.50424	-59.4022875	43.73143933
EDDM	-1699.370867	-1327.567232	-62.7970625	46.2306427
HIDA	-1342.886513	-894.69254	-23.93787	17.62284844
HIDS	-1719.248956	-1347.466375	-49.5856775	36.50453776
EDDS	-1777.980573	-1406.172229	-65.75196	48.40601213
IDS	-1644.005468	-1272.221386	-50.527555	37.19793966
IDA	-1189.035561	-777.551303	-25.2713075	18.60451335
PDA	-1302.204625	-664.387198	-28.38308	20.89537277

**G H2O**	**-76.452265**
**G Ca(H2O)6**	**-1136.22621**

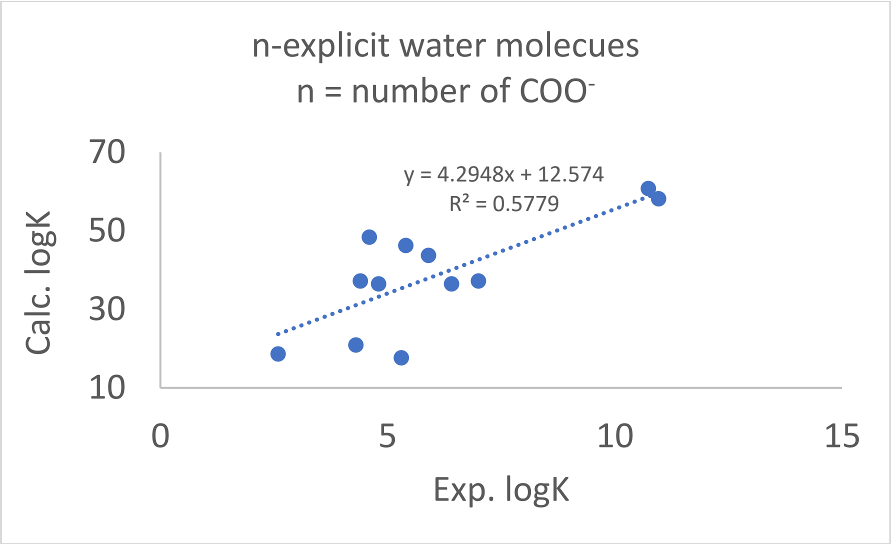

Figure S3. Correlation between calculate and experimental logK values with explicit water molecules equal to the number of COO^- functional. As can be seen R^2 slightly improved (0.07 units) with respect to the case with 6 water molecules for all ligands.

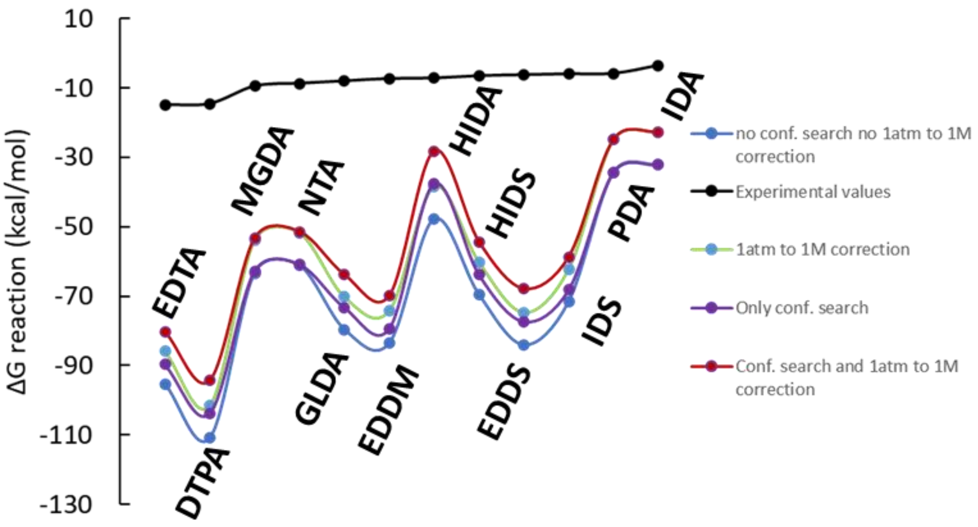

Figure S4. Comparison of the calculated ΔG values for method i without explicit hydration of ligand vs. experimental ΔG. Various cases including conformational search and 1atm to 1M correction are compared.

Table S16. Energies (in ha) of new conformers using CREST method for the large free ligand molecules with various dihedral angles. The bold values show the lowest energy conformers.

Ligand	Original energy based on optimized geometry	conf.1	conf. 2	conf. 3	conf. 4	conf. 5	conf. 6	conf. 7	conf. 8	conf. 9	conf. 10	conf. 11	conf. 12	conf. 13
EDTA	**-1100.306198**	-1100.3089	-1100.3108	-1100.315	-1100.315081	**-1100.315164**	-1100.315174	-1100.312417	-1100.312098	-1100.309431	-1100.309685	-1100.309692		
DTPA	**-1461.705182**	-1461.7134	**-1461.7167**	-1461.7162	-1461.712069	-1461.715565	-1461.712556	-1461.711618	-1461.715012	-1461.715466				
MGDA	**-778.216785**	-778.2161	-778.21353	-778.214195	-778.216213	-778.212284	-778.213499	-778.215766	-778.213588	-778.216285	-778.216352	-778.208169	-778.208	**-778.217713**
NTA	**-738.91498**	-738.91417	**-738.91552**	-738.91416	-738.910891	-738.914174	-738.910891	-738.915046	-738.914945					
GLDA	**-1005.667735**	-1005.6679	-1005.6688	-1005.6715	**-1005.673094**	-1005.673043	-1005.670761	-1005.670806	-1005.671	-1005.671356				
EDDM	**-1021.725003**	-1021.7296	-1021.7301	-1021.7313	-1021.730057	**-1021.731847**	-1021.731277	-1021.730417	-1021.731174					
HIDA	**-665.297644**	-665.31321	**-665.31389**	-665.30927	-665.311822	-665.311815	-665.311736							
HIDS	**-1041.631767**	-1041.6302	-1041.63	-1041.6302	-1041.627261	-1041.631939	-1041.632372	-1041.63241	-1041.626047	-1041.627	-1041.633097	**-1041.634626**		
EDDS	**-1100.333906**	-1100.3329	-1100.3391	-1100.3353	-1100.334229	-1100.33346	-1100.3426	-1100.341926	-1100.34231	-1100.342147	**-1100.344758**	-1100.342302		
IDS	**-966.378729**	-966.37974	-966.38418	-966.37777	-966.383221	-966.384182	**-966.384242**	-966.377458						

XYZ coordinates of chelators associated with Na^+ (at b3lyp/6-311g opt freq SCRF=(PCM,solvent=H2O) em=gd3bj)**

EDTA-4Na

G = -1749.510426 ha

C -0.011989 -1.456069 1.333933
C -0.567321 -0.110830 1.809433
N -0.078266 1.027906 1.017566
C 1.132320 1.644548 1.586718
C 2.419275 0.853058 1.306159
O 2.795141 -0.011635 2.133749
C -1.083066 2.064430 0.773920
C -0.768291 2.938972 -0.469700
O -0.163739 2.356154 -1.433184
O -1.170236 4.111717 -0.451783
O 3.027355 1.114089 0.222324
N -0.501790 -1.886626 0.015743
C -1.827142 -2.495501 0.082996
C -2.988348 -1.483441 0.061976
C 0.503015 -2.693464 -0.681429
C 1.654525 -1.811775 -1.221135
O 2.828280 -2.158034 -0.950370
O 1.317196 -0.800683 -1.904143
O -4.018313 -1.765135 0.716618
O -2.853996 -0.438357 -0.648516
H -0.249003 -2.219971 2.093718
H 1.072120 -1.380294 1.294430
H -0.300710 0.008476 2.870264
H -1.656575 -0.126058 1.764045
H 1.021119 1.779046 2.673889
H 1.250954 2.627607 1.130936
H -1.226880 2.730611 1.637319
H -2.039479 1.572984 0.574224
H 0.029025 -3.169837 -1.543705
H 0.925084 -3.484486 -0.045989
H -1.946998 -3.141105 0.964261
H -1.971042 -3.123133 -0.801201
Na 1.910172 1.429208 -1.707905
Na -4.936612 0.333109 0.103554
Na -0.773749 0.089669 -1.418457
Na 4.189407 -0.945378 0.402795

DTPA-5Na

G = -2273.223044 ha

N 2.716695 -1.311381 0.531499
C 2.195285 -2.449903 1.288097
C 0.959973 -2.085998 2.110318
N -0.249043 -1.791680 1.320756
C 3.459187 -0.335382 1.346507
C 2.928559 1.096128 1.147818
O 3.722081 2.005400 0.773528
O 1.698727 1.272983 1.364638
C 3.453403 -1.679869 -0.677047
C 3.690460 -0.425503 -1.539846
O 2.678071 0.305714 -1.767342
O 4.859951 -0.158174 -1.901297
C -1.311411 -1.269873 2.205657
C -1.551536 0.236431 2.068455
H -2.238686 -1.810205 2.009090
H -1.062024 -1.481048 3.252716
C -0.655507 -2.942837 0.500643
C -0.106026 -2.899294 -0.948020
O 0.445810 -3.915700 -1.402713
O -0.293117 -1.798576 -1.565422
H 2.951844 -2.862648 1.974923
H 1.948813 -3.241817 0.580183
H 1.181744 -1.202701 2.712933
H 0.762901 -2.918381 2.808634
H 4.530532 -0.372141 1.123434
H 3.333877 -0.573537 2.405686
H -0.372680 -3.896126 0.966851
H 2.847061 -2.390312 -1.245451
H 4.424269 -2.145267 -0.452882
H -1.744218 -2.936639 0.406263
N -2.448758 0.597426 0.954248
C -3.841502 0.274044 1.261805
C -4.778716 0.241933 0.044136
C -2.320939 2.025460 0.640892
C -1.059352 2.375354 -0.170248
O -0.682310 1.532432 -1.035110
O -0.518974 3.481944 0.062460
O -6.007980 0.287728 0.282933
O -4.275233 0.137341 -1.116064
H -3.893290 -0.724901 1.701241
H -2.346898 2.644285 1.549098
H -0.588138 0.724197 1.910407
H -1.958277 0.626403 3.017641
H -3.172484 2.320142 0.020980
H -4.271264 0.961286 2.006788
Na 1.698681 3.471536 0.580528
Na 4.204298 2.135018 -1.510733
Na -6.452630 0.152231 -2.033478
Na 0.803812 -0.081519 -0.388588
Na -2.058964 -0.345812 -1.341724

MGDA-3Na

G = -1265.091451 ha

N -0.653221 -0.016183 1.409697
C -0.280474 1.384532 1.519002
C 1.033559 1.692394 0.774888
C -1.857379 -0.414177 0.681975
C -1.798502 0.145423 -0.758423
C 0.278622 -1.042104 1.846054
C 0.836887 -1.847049 0.654682
O 0.993059 1.860677 -0.484480
O 2.097731 1.668213 1.443551
O -1.144828 -0.516753 -1.625305
O -2.309581 1.271093 -0.980918
O 0.257132 -2.924573 0.364267
O 1.771982 -1.338889 -0.039125
H -0.148505 1.670013 2.571160
H -1.085854 1.980414 1.091431
H -1.814829 -1.500712 0.602626
H 1.095290 -0.550597 2.375095

H	-0.215654	-1.744692	2.529655
C	-3.135227	-0.009559	1.419550
H	-4.023193	-0.346805	0.875971
H	-3.199368	1.075025	1.519343
H	-3.146008	-0.456579	2.417060
Na	2.988213	0.505821	-0.417527
Na	0.391493	-2.133740	-1.870746
Na	-0.350451	1.661765	-2.266319

NTA-3Na

G = -1225.787505 ha

N	-0.007007	0.001007	0.372841
C	-1.173080	-0.747584	0.823211
C	-2.520423	-0.366895	0.188204
C	1.222975	-0.631850	0.828864
C	1.571271	-1.988618	0.196509
C	-0.070321	1.384851	0.822806
C	0.939896	2.355186	0.189732
O	-2.564214	0.472680	-0.749717
O	-3.527230	-0.962099	0.668748
O	0.875541	-2.446323	-0.748423
O	2.587475	-2.560458	0.685018
O	1.666555	1.976855	-0.767330
O	0.953259	3.517504	0.687034
H	-1.303417	-0.685580	1.920905
H	-1.012563	-1.800542	0.585718
H	1.233050	-0.773562	1.926825
H	2.052675	0.034842	0.589963
H	-1.059669	1.773728	0.576903
H	0.042317	1.467625	1.921141
Na	-4.892915	0.228135	-0.805159
Na	2.268704	-4.327362	-0.810055
Na	2.655452	4.095648	-0.805689

GLDA-4Na

G = -1654.850040 ha

N	1.119120	0.792342	0.865450
C	0.211921	1.786858	1.431365
C	-0.978396	2.134119	0.516215
O	-0.788450	2.113679	-0.738339
O	-2.066245	2.430567	1.065870
C	2.541439	1.010753	1.192262
C	3.489830	0.840038	-0.025645
C	0.745743	-0.611226	1.145487
C	1.571981	-1.547557	0.233858
O	2.125096	-2.542187	0.760425
O	1.665054	-1.230954	-0.989798
O	3.237177	1.546350	-1.035767
O	4.442454	0.023416	0.094110
H	0.764666	2.721254	1.556143
H	-0.173405	1.512386	2.422286
H	1.029485	-0.850663	2.181729
H	2.868831	0.345908	2.001308
H	2.668824	2.039021	1.536359
C	-0.753697	-0.948041	1.024355
C	-1.348266	-1.073338	-0.388918
C	-2.861386	-1.254400	-0.301815
O	-3.307611	-2.315968	0.199057
O	-3.605649	-0.303176	-0.712121
H	-1.329450	-0.215071	1.594525
H	-0.899667	-1.903275	1.534324
H	-0.917639	-1.941205	-0.891955
H	-1.131829	-0.188378	-0.980065
Na	3.941683	-2.051030	-0.744134
Na	1.069612	0.986876	-1.589435
Na	-3.160004	1.896147	-0.976843
Na	-5.491589	-1.455941	0.117386

EDDM-4Na

G = -1670.911925 ha

N	-1.267231	1.117071	0.768831
C	-0.797677	1.074125	2.161717
C	0.695682	1.378110	2.222665
N	1.493489	0.259041	1.696940
C	2.594880	0.643431	0.820869
C	2.172069	1.532687	-0.399287
H	3.335600	1.210842	1.384613
C	3.285446	-0.606501	0.250698
O	2.533505	-1.554112	-0.143915
O	4.535296	-0.613384	0.163378
O	2.849983	2.535607	-0.661366
O	1.129820	1.139355	-1.032596
C	-2.426584	0.268395	0.516957
C	-3.057624	0.541264	-0.881208
H	-3.194572	0.474441	1.265279
C	-2.091299	-1.257941	0.578062
O	-0.901143	-1.578741	0.307962
O	-3.025023	-2.049480	0.853807
O	-2.358452	1.155760	-1.726608
O	-4.217645	0.091235	-1.067292
H	-0.508236	0.741922	0.202850
H	-1.349991	1.808642	2.755895
H	-0.968785	0.089331	2.623692
H	0.979849	1.584455	3.260774
H	0.882395	2.302143	1.656808
H	0.898527	-0.409357	1.216799
Na	0.605659	-1.114231	-1.315469
Na	4.436260	-2.721736	-0.892466
Na	-5.126447	-1.640428	0.082897
Na	-0.668079	2.577617	-1.220135

HIDA-2Na (with OH)

G = -989.884880 ha

N	0.018906	1.400792	0.889150
C	0.204834	0.341887	1.868631
C	0.887563	-0.885831	1.235379
O	0.171717	-1.720462	0.601731
O	2.144197	-0.932248	1.285643
C	-1.322322	1.716474	0.449754
C	-1.989870	0.518182	-0.257298
O	-2.908406	-0.086644	0.341335
O	-1.499890	0.142405	-1.374018
H	2.263920	2.108064	-1.603921
H	-0.776322	0.064060	2.256378
H	0.824661	0.691555	2.704407
H	-1.273577	2.562812	-0.239701

H	-1.956368	2.004381	1.297719
O	1.102408	0.420028	-1.649094
H	0.125282	0.256107	-1.563359
C	1.277718	1.779175	-1.263243
C	1.177696	1.993462	0.255845
H	2.057275	1.553949	0.727917
H	1.218731	3.082111	0.433156
H	0.531208	2.398143	-1.776555
Na	2.082212	-1.571599	-0.969268
Na	-1.776854	-2.054896	-0.470665

HIDA-3Na (with O$^-$)

G = -1151.677092 ha

N	0.049394	0.893170	0.805461
C	-0.073566	-0.314320	1.631363
C	0.416957	-1.511929	0.794831
O	-0.329384	-1.859209	-0.173230
O	1.543726	-1.999603	1.054718
C	-1.157945	1.712744	0.744240
C	-2.228155	0.904777	-0.013676
O	-3.151144	0.370591	0.647907
O	-2.030136	0.717707	-1.254440
H	2.885448	2.512667	-0.186333
H	-1.122928	-0.466024	1.889724
H	0.506831	-0.232737	2.557061
H	-0.925918	2.619976	0.182784
H	-1.531987	1.995807	1.740266
O	2.230905	0.775048	-1.146461
C	1.972675	1.918064	-0.413795
C	1.311278	1.627498	0.947881
H	1.998696	1.006261	1.529439
H	1.161692	2.562380	1.514849
H	1.302775	2.628776	-0.952152
Na	3.120871	-1.076690	-0.335594
Na	-2.570713	-1.594566	-0.667194
Na	0.160791	0.040659	-1.641474

HIDS-4Na

G = -1690.803433 ha

N	0.201192	-0.867912	-0.877068
C	1.377978	-0.034502	-1.072797
C	1.652911	0.832798	0.181146
O	2.060398	2.017284	0.010998
O	1.455850	0.311880	1.314028
C	2.621703	-0.916537	-1.328533
C	2.942181	-1.921235	-0.190544
O	2.018798	-2.743853	0.113374
O	4.072566	-1.854854	0.330700
C	-1.118330	-0.560050	-1.376087
C	-2.137915	-1.556763	-0.750417
O	-3.329154	-1.447188	-1.123756
O	-1.721974	-2.355086	0.139281
C	-1.626138	0.870352	-1.087563
C	-1.550447	1.287254	0.398840
O	-0.918220	2.340086	0.685435
O	-2.153952	0.545882	1.208491
H	0.425922	-1.844534	-1.016676
H	-1.207986	-0.676124	-2.471897
H	-2.685820	0.860733	-1.354325
H	3.488195	-0.271871	-1.481900
H	2.449013	-1.478602	-2.253326
O	-0.946107	1.847794	-1.892454
H	1.261508	0.643651	-1.922118
H	-1.036409	1.587416	-2.815584
Na	0.265445	-1.721929	1.351762
Na	-3.500079	-1.294434	1.385050
Na	0.828325	2.348068	2.226634
Na	0.462668	3.391137	-0.915970

EDDS-4Na

G = -1749.531544 ha

N	-0.834299	1.447805	-0.158674
C	-0.161578	2.688281	-0.556155
C	1.273106	2.723976	-0.027944
N	2.051825	1.637408	-0.618871
C	3.209898	1.102236	0.096547
C	3.740095	-0.154873	-0.646088
C	2.859014	0.711943	1.545982
C	1.607740	-0.163129	1.647392
O	1.544949	-1.195136	0.894181
O	0.694189	0.172247	2.433104
O	4.704549	-0.753188	-0.108097
O	3.132391	-0.544183	-1.684060
C	-2.232244	1.362053	-0.594270
C	-3.165766	0.849978	0.516813
C	-2.397016	0.390355	-1.804016
C	-2.302079	-1.072221	-1.341314
O	-3.378000	-1.688745	-1.136702
O	-1.144677	-1.539676	-1.109196
O	-4.389625	1.084541	0.390248
O	-2.654154	0.116030	1.416417
H	-0.795798	1.350353	0.852077
H	-0.695842	3.583388	-0.199193
H	-0.148773	2.743628	-1.650961
H	1.239772	2.591240	1.053908
H	1.706574	3.716703	-0.215331
H	2.331196	1.880634	-1.562774
H	4.047804	1.814934	0.150898
H	2.726493	1.593008	2.172977
H	3.712209	0.151407	1.932641
H	-3.372900	0.559812	-2.258821
H	-1.620730	0.621702	-2.537719
H	-2.601432	2.344626	-0.907731
Na	-4.661853	-1.275009	0.731235
Na	-0.838860	-1.315372	1.293308
Na	3.098274	-2.575076	-0.129376
Na	0.791960	-0.324965	-1.274803

IDS-4Na

G = -1615.561451 ha

N	0.114096	0.734947	0.742935
C	-1.210923	1.349970	0.759587
C	-2.016714	1.317127	-0.555509
O	-2.952236	2.148216	-0.636149
O	-1.809869	0.387128	-1.389749
C	-2.064296	0.571383	1.804296

C -2.407855 -0.833078 1.282941
O -3.533384 -0.989169 0.740465
O -1.511461 -1.727613 1.349435
C 1.180860 1.341767 -0.058300
C 2.528769 1.130530 0.661019
O 3.493739 1.848915 0.315268
O 2.598454 0.148122 1.464818
C 1.332163 0.686348 -1.465185
C 1.915108 -0.733203 -1.371325
O 3.154186 -0.870923 -1.528441
O 1.119049 -1.680896 -1.079078
H 0.441219 0.758595 1.704285
H 1.023612 2.418786 -0.201067
H 2.013038 1.307230 -2.047932
H -2.981710 1.127843 1.990922
H -1.495336 0.502261 2.735583
H -1.168035 2.402988 1.071475
H 0.350913 0.663893 -1.935077
Na 0.850627 -1.562630 1.282623
Na -1.146685 -1.745088 -1.043296
Na -4.257520 0.154096 -1.136355
Na 4.645137 -0.248336 0.100990

PDA-2Na

G = -949.198434 ha
N -0.000008 0.125562 -0.000509
C -1.151450 -0.556934 0.012284
C -1.195119 -1.954378 0.021660
C 0.000218 -2.662945 0.000267
C 1.195414 -1.954222 -0.021433
C 1.151477 -0.556765 -0.012860
C 2.459897 0.235603 -0.012660
O 3.521834 -0.436167 -0.145356
O 2.404931 1.483376 0.125398
C -2.459958 0.235363 0.011972
O -3.521856 -0.436392 0.145428
O -2.405184 1.483110 -0.126595
H -2.156764 -2.446860 0.043584
H 0.000302 -3.747032 0.000548
H 2.157156 -2.446547 -0.042851
Na -4.746548 1.558934 -0.041193
Na 4.746429 1.558862 0.042640

IDA-2Na

G = -836.036143 ha
N -0.000034 0.670418 -0.423841
C -1.222509 1.286297 0.063125
C -2.412402 0.316795 0.018216
O -2.160735 -0.920153 0.027268
O -3.567893 0.820934 0.017015
C 1.222481 1.286300 0.063006
C 2.412386 0.316801 0.017994
O 2.160726 -0.920149 0.027222
O 3.567877 0.820935 0.016505
H -0.000013 -0.301534 -0.127683
H -1.479153 2.179859 -0.514224
H -1.136991 1.616582 1.117764
H 1.137083 1.616562 1.117665
H 1.479035 2.179875 -0.514358
Na 4.469704 -1.346984 0.009725
Na -4.469637 -1.347011 0.009328

XYZ coordinates of chelators associated with Na^+ and complexed to Ca^{2+} (at b3lyp/6-311g opt freq SCRF=(PCM, solvent=H2O) em=gd3bj)**

EDTA-Ca-2Na

G = -2102.531723 ha
C -0.241973 0.724157 2.204371
C 0.242123 -0.723948 2.204409
N -0.236662 -1.475547 1.029676
C -1.652742 -1.848996 1.135649
C -2.400964 -1.816334 -0.206799
O -3.642899 -1.920391 -0.159058
C 0.617310 -2.639559 0.756630
C 1.977234 -2.264430 0.132807
O 2.952260 -2.983360 0.362852
O 1.964523 -1.228134 -0.626831
O -1.727824 -1.606264 -1.266827
N 0.236685 1.475663 1.029519
C 1.652779 1.849133 1.135330
C 2.400927 1.816328 -0.207173
C -0.617332 2.639648 0.756477
C -1.977388 2.264519 0.132906
O -2.952256 2.983674 0.362887
O -1.964921 1.228039 -0.626499
O 3.642871 1.920398 -0.159483
O 1.727756 1.606190 -1.267150
H 0.064994 1.208627 3.142208
H -1.333076 0.745278 2.186490
H -0.064747 -1.208331 3.142325
H 1.333225 -0.745079 2.186416
H -2.169879 -1.136498 1.777752
H -1.786710 -2.835395 1.599239
H 0.103554 -3.269948 0.025881
H 0.785341 -3.244854 1.656708
H -0.103678 3.269967 0.025595
H -0.785219 3.245036 1.656519
H 2.169963 1.136713 1.777483
H 1.786768 2.835582 1.598806
Ca -0.000135 0.000099 -1.058719
Na -3.679565 0.033806 -1.602336
Na 3.680117 -0.034554 -1.601498

DTPA-Ca-3Na

G = -2626.242318 ha
N 1.425086 -1.498376 1.392769
C 0.583869 -1.670382 2.584742
C -0.145979 -0.393241 2.997863
N -1.223140 -0.001408 2.063214
C 2.707990 -0.841475 1.683363
C 2.768996 0.637782 1.292192
O 1.672641 1.227895 1.046111
O 3.897344 1.170752 1.211389
C 1.619094 -2.734699 0.634286
C 2.061897 -2.457114 -0.808314
O 1.686345 -1.353942 -1.334659

O	2.733108	-3.320315	-1.405844
C	-1.723183	1.365116	2.359199
C	-1.322351	2.434676	1.335752
H	-2.809896	1.334527	2.443202
H	-1.361502	1.699680	3.337159
C	-2.301845	-1.008973	2.027524
C	-2.340705	-1.883537	0.764503
O	-3.399221	-2.499723	0.526766
O	-1.310289	-1.902651	0.021783
H	1.182936	-2.012870	3.442219
H	-0.141944	-2.454631	2.374252
H	0.564032	0.430707	3.047774
H	-0.560692	-0.546648	4.006867
H	3.513557	-1.343688	1.145432
H	2.959642	-0.918281	2.749491
H	-2.245477	-1.680545	2.893767
H	0.655808	-3.243166	0.560540
H	2.338399	-3.420605	1.100586
H	-3.275033	-0.524192	2.096172
N	-1.533105	2.033297	-0.065009
C	-2.885345	1.588492	-0.405556
C	-2.891500	0.505454	-1.503019
C	-1.075287	3.078978	-0.994355
C	0.445026	3.045738	-1.206637
O	0.937069	1.929448	-1.566120
O	1.107813	4.086313	-1.004690
O	-4.001456	0.036155	-1.830494
O	-1.770953	0.098524	-1.939418
H	-3.362357	1.133034	0.460381
H	-1.383972	4.075653	-0.655494
H	-0.258191	2.654294	1.440548
H	-1.875990	3.355775	1.581122
H	-1.533138	2.890042	-1.966745
H	-3.538940	2.416617	-0.710499
Ca	0.066367	0.028279	-0.314566
Na	-2.771240	-2.134377	-1.887873
Na	2.943466	2.704786	-0.391876
Na	2.891434	-1.836446	-3.280261

MGDA-Ca-Na

G = -1618.104531 ha

N	0.534079	-0.103108	0.645944
C	0.430330	1.170512	1.366392
C	1.051613	2.356065	0.589969
C	-0.534461	-1.067541	0.978574
C	-1.860486	-0.624638	0.320550
C	1.875179	-0.690796	0.733173
C	2.201497	-1.633992	-0.447699
O	1.096457	2.232921	-0.684209
O	1.409584	3.340096	1.245115
O	-1.772711	-0.013860	-0.804418
O	-2.941167	-0.904786	0.865772
O	3.049318	-2.513087	-0.265384
O	1.585151	-1.385814	-1.543479
H	0.874413	1.132384	2.367255
H	-0.628600	1.414411	1.495665
H	-0.271977	-1.994196	0.454842
H	2.603938	0.123945	0.687471
H	2.050103	-1.222619	1.675338
Ca	0.249399	0.446042	-1.819295
C	-0.690362	-1.382011	2.467272
H	-1.425615	-2.174593	2.602300
H	-1.033949	-0.508796	3.024643
H	0.257514	-1.715749	2.894708
Na	-4.127754	0.176071	-0.899500

NTA-Ca-Na

G = -1578.798625 ha

N	-0.534339	0.001022	-0.799141
C	0.566470	-0.730299	-1.427048
C	1.914495	-0.444292	-0.753914
C	-1.825052	-0.674680	-0.969072
C	-2.010369	-1.871578	-0.006981
C	-0.577479	1.410093	-1.204393
C	-1.242121	2.320437	-0.145069
O	1.884892	-0.096127	0.479463
O	2.959816	-0.601034	-1.406846
O	-1.367469	-1.800349	1.099533
O	-2.786403	-2.768736	-0.349287
O	-1.736968	3.383576	-0.531498
O	-1.174661	1.905794	1.065293
H	0.648786	-0.533957	-2.503088
H	0.388447	-1.803191	-1.307447
H	-1.990464	-1.006921	-2.001549
H	-2.618164	0.039299	-0.728520
H	0.449950	1.770125	-1.315737
H	-1.075436	1.551233	-2.171748
Ca	-0.090443	-0.001689	1.694069
Na	4.232435	-0.021186	0.538752

GLDA-Ca-2Na (four coordinated to Ca^{2+})

G = -2007.865941 ha

N	-0.135891	-0.386460	1.002473
C	1.130777	-0.705741	1.674106
C	2.001221	-1.682140	0.857409
O	1.879466	-1.602852	-0.422839
O	2.802978	-2.394383	1.466673
C	-1.104414	-1.490495	1.245139
C	-2.080213	-1.714767	0.070351
C	-0.755727	0.911105	1.371526
C	-1.854135	1.248537	0.340941
O	-2.903025	1.786235	0.738451
O	-1.617427	0.934263	-0.874302
O	-1.537837	-2.035943	-1.032631
O	-3.302802	-1.555847	0.266486
H	0.980868	-1.103350	2.684313
H	1.740622	0.192239	1.758742
H	-1.256225	0.807493	2.342960
H	-1.663106	-1.313000	2.170745
H	-0.534931	-2.414155	1.360116
C	0.182215	2.129642	1.502459
C	0.832854	2.692950	0.225419
C	1.918035	1.804084	-0.374124
O	1.661483	1.227002	-1.489617
O	2.992174	1.653295	0.244768

H	0.951593	1.932430	2.250938
H	-0.446814	2.918066	1.924010
H	1.304165	3.642035	0.494180
H	0.072629	2.892365	-0.529319
Ca	0.130748	-0.511567	-1.562760
Na	3.632001	-0.156657	-1.194925
Na	-4.017117	0.350223	-0.884535

GLDA-Ca-2Na (three coordinated to Ca^{2+})

G = -2007.84038 ha

N	-1.332958	-0.044836	0.624813
C	-0.959586	-1.176173	1.484468
C	-0.049885	-2.213564	0.789470
O	-0.233114	-2.348287	-0.472767
O	0.738658	-2.859574	1.487333
C	-2.688925	0.432799	0.946009
C	-3.812190	-0.496411	0.434993
C	-0.390788	1.102483	0.626177
C	-0.586768	1.923607	-0.673096
O	-0.250542	3.126813	-0.651539
O	-1.023350	1.303550	-1.689253
O	-3.542049	-1.164295	-0.624070
O	-4.886355	-0.490221	1.044529
H	-1.877328	-1.716871	1.733566
H	-0.513199	-0.861868	2.433726
H	-0.633580	1.757010	1.474654
H	-2.843960	1.395885	0.450977
H	-2.817284	0.600249	2.022911
C	1.105915	0.750747	0.765110
C	1.809785	0.213746	-0.479679
C	3.267981	-0.200094	-0.248865
O	3.873395	-0.702718	-1.238398
O	3.781440	-0.030141	0.891970
H	1.244498	0.049095	1.587250
H	1.611185	1.667045	1.076962
H	1.808131	0.960762	-1.279397
H	1.304135	-0.665506	-0.880022
Ca	-1.561628	-0.960073	-1.723087
Na	5.747680	-1.037611	0.115504
Na	0.565298	4.639944	0.758810

EDDM-Ca-2Na

G = -2023.922821 ha

N	1.027980	-0.991504	1.163455
C	0.739429	-0.192003	2.361313
C	-0.739623	0.194107	2.361186
N	-1.028266	0.992700	1.162720
C	-2.423756	1.046609	0.722773
C	-2.894789	-0.354556	0.290362
H	-3.117635	1.440032	1.470407
C	-2.503228	1.933883	-0.570148
O	-1.529976	1.771178	-1.378404
O	-3.485730	2.668953	-0.709549
O	-4.083033	-0.680656	0.470564
O	-2.025636	-1.089141	-0.295022
C	2.423470	-1.046193	0.723658
C	2.502766	-1.934458	-0.568591
H	3.117183	-1.439256	1.471637
C	2.894940	0.354528	0.290258
O	2.026166	1.088758	-0.296147
O	4.083144	0.680603	0.470749
O	3.485233	-2.669663	-0.707528
O	1.529338	-1.772485	-1.376782
H	0.676925	-1.935516	1.292242
H	0.997253	-0.714962	3.290983
H	1.345760	0.716057	2.320773
H	-0.997426	0.717773	3.290459
H	-1.345935	-0.713993	2.321365
H	-0.677612	1.936937	1.290971
Ca	0.000054	-0.000113	-0.922030
Na	3.696330	2.653509	-0.803866
Na	-3.695321	-2.653969	-0.803349

HIDA-Ca (OH protonated)

G = -1342.886513

N	0.053210	-0.016047	-0.821714
C	-0.696425	-1.276174	-0.933056
C	-1.968825	-1.309355	-0.056991
O	-1.924713	-0.597962	1.008717
O	-2.896614	-2.039640	-0.414775
C	1.467822	-0.203839	-1.165105
C	2.281925	-0.827007	-0.006656
O	1.794422	-0.668092	1.168040
O	3.344903	-1.385254	-0.291443
H	-0.766957	3.191832	-1.642134
H	-0.054229	-2.086395	-0.576303
H	-0.961460	-1.504636	-1.972891
H	1.921232	0.769242	-1.369675
H	1.597005	-0.811507	-2.069015
Ca	-0.070020	0.540714	1.658307
O	-0.442702	2.508823	0.302158
H	-0.275366	3.405630	0.611454
C	-0.181901	2.434111	-1.116028
C	-0.605093	1.055958	-1.585559
H	-1.681451	0.959349	-1.430034
H	-0.417077	0.966756	-2.665094
H	0.878853	2.622623	-1.301657

HIDA-Ca-Na (O^- deprotonated)

G = -1504.685411

N	-0.637883	-0.121010	0.829046
C	-1.364186	-1.383237	0.673630
C	-0.606907	-2.434637	-0.171027
O	0.194435	-1.970550	-1.055002
O	-0.863527	-3.626919	0.033040
C	-1.541491	1.007297	1.061736
C	-2.151916	1.571539	-0.244084
O	-1.464953	1.389153	-1.309159
O	-3.220317	2.188277	-0.155664
H	2.332617	0.661399	2.303417
H	-2.297470	-1.178448	0.139116
H	-1.637384	-1.826714	1.640781
H	-0.975183	1.826425	1.512478
H	-2.350421	0.757560	1.760294
Ca	0.553204	0.278740	-1.357020
O	1.985252	0.853176	0.256098

C	1.525573	0.854925	1.571955
C	0.459024	-0.223992	1.807435
H	0.930045	-1.199587	1.659149
H	0.081713	-0.189834	2.843453
H	1.106939	1.839132	1.855511
Na	4.112959	0.687806	-0.248136

HIDS-Ca-2Na

G = -2043.810449 ha

N	0.037390	-0.617023	0.994136
C	-1.336038	-0.249199	1.353631
C	-1.967204	0.641077	0.265410
O	-2.753549	1.547480	0.599265
O	-1.650726	0.371317	-0.943847
C	-2.187089	-1.531957	1.466376
C	-2.325255	-2.390335	0.180827
O	-1.262802	-2.608645	-0.503594
O	-3.449626	-2.840705	-0.076959
C	1.200157	0.185075	1.375650
C	2.442651	-0.473837	0.726403
O	3.567662	-0.060889	1.067589
O	2.240576	-1.396618	-0.133088
C	1.142390	1.667592	0.970306
C	0.771060	1.928579	-0.510617
O	0.049515	2.935398	-0.713182
O	1.216093	1.129560	-1.376677
H	0.212508	-1.570347	1.289754
H	1.367401	0.184421	2.461115
H	2.166079	2.041664	1.101318
H	-3.189953	-1.262339	1.797616
H	-1.749008	-2.166651	2.246565
O	0.259047	2.380704	1.826121
H	-1.381139	0.296626	2.298801
Ca	0.195398	-1.059461	-1.396649
H	-0.235852	2.949827	1.212242
Na	4.524846	-1.289983	-0.726455
Na	-2.118779	2.627474	-1.488667

EDDS-Ca-2Na

G = -2102.538603 ha

N	1.285710	-1.434122	0.503817
C	0.762218	-2.605210	-0.209924
C	-0.762277	-2.608854	-0.102444
N	-1.281117	-1.358740	-0.670195
C	-2.697268	-1.027572	-0.446172
C	-2.960833	0.426514	-0.897650
C	-3.140953	-1.222060	1.010436
C	-2.282945	-0.562802	2.112525
O	-1.497939	0.383140	1.762610
O	-2.437650	-0.985796	3.267106
O	-4.143630	0.822861	-0.907293
O	-1.962584	1.147067	-1.234636
C	2.700953	-1.078576	0.314884
C	2.968045	0.309656	0.938751
C	3.136461	-1.090858	-1.156958
C	2.270352	-0.299695	-2.161671
O	1.499691	0.604740	-1.689917
O	2.404748	-0.585765	-3.360304
O	4.151208	0.703590	0.982091
O	1.973444	0.981327	1.375423
H	1.123197	-1.561065	1.498879
H	1.166193	-3.554742	0.166223
H	1.042435	-2.530190	-1.261867
H	-1.042508	-2.660044	0.950868
H	-1.169304	-3.504792	-0.590362
H	-1.113624	-1.363222	-1.672458
H	-3.358272	-1.653666	-1.062007
H	-3.234575	-2.283239	1.247707
H	-4.147343	-0.803524	1.091217
H	4.142280	-0.664166	-1.191417
H	3.228593	-2.113999	-1.524862
H	3.364261	-1.776096	0.845578
Ca	0.001565	0.700397	0.050500
Na	-3.387176	3.009751	-1.441118
Na	3.393938	2.808274	1.776195

IDS-Ca-2Na

G = -1968.568184 ha

N	-0.315550	-0.789490	1.108453
C	-1.519975	0.018337	1.382799
C	-1.536888	1.253595	0.459686
O	-1.888748	2.357485	0.906511
O	-1.127992	1.074196	-0.748469
C	-2.788146	-0.839950	1.143353
C	-3.048881	-1.431596	-0.281282
O	-2.008890	-1.872862	-0.922570
O	-4.204717	-1.475531	-0.662793
C	1.027155	-0.416234	1.583654
C	2.058375	-1.353775	0.898838
O	3.244716	-1.251436	1.262126
O	1.666387	-2.051855	-0.102886
C	1.435084	1.044614	1.263953
C	1.598622	1.471733	-0.218372
O	1.470205	2.678741	-0.475619
O	1.875783	0.560074	-1.085460
H	-0.495791	-1.755709	1.360833
H	1.130933	-0.537258	2.669999
H	2.410418	1.197797	1.734876
H	-3.663489	-0.246202	1.407661
H	-2.760341	-1.685521	1.843151
H	-1.543820	0.387952	2.414871
Ca	-0.094421	-0.938354	-1.258248
H	0.752190	1.746829	1.744587
Na	3.602289	-0.924834	-0.944453
Na	-0.629459	3.338906	-0.740338

IDA-Ca

G = -1189.035561 ha

N	-0.000158	-0.833678	0.476071
C	1.269862	-1.373950	-0.074915
C	2.417251	-0.323183	-0.077655
O	2.002561	0.913951	-0.152572
O	3.565481	-0.709741	-0.053709
C	-1.269792	-1.373968	-0.075771
C	-2.417252	-0.323294	-0.077923
O	-2.002355	0.913738	-0.153922

O	-3.565530	-0.709567	-0.052524
H	-0.000513	-1.002873	1.478823
H	1.096650	-1.650646	-1.119337
H	1.610781	-2.273242	0.443773
H	-1.610578	-2.273799	0.442069
H	-1.096098	-1.649644	-1.120385
Ca	-0.000040	1.589263	0.084097

PDA-Ca

G = -1302.204625 ha

N	0.000017	0.140845	0.000128
C	-1.172958	0.778009	-0.000041
C	-1.213379	2.168462	-0.000123
C	0.000142	2.861037	0.000041
C	1.213542	2.168411	0.000205
C	1.172961	0.777891	0.000228
C	2.390393	-0.162816	0.000637
O	3.508374	0.312735	-0.001187
O	2.050061	-1.421740	0.000260
C	-2.390391	-0.162579	-0.000717
O	-3.508380	0.312864	0.000544
O	-2.050063	-1.421620	0.000649
H	-2.171475	2.671691	-0.000411
H	0.000117	3.945605	0.000015
H	2.171757	2.671413	0.000376
Ca	-0.000116	-2.155151	-0.000219

$Ca^{2+}(H_2O)_6$

G = -1136.22621 ha

Ca	0.008460	-0.082452	-0.028789
O	1.831907	-1.511626	0.640556
O	-1.862393	-0.900371	-1.338402
O	-1.645929	1.668837	-0.128911
O	1.661006	1.309671	1.030736
O	1.799136	0.249652	-1.618931
O	-1.813283	-0.666923	1.462440
H	1.836486	-2.321389	1.162530
H	-1.888024	-1.345082	-2.192465
H	-2.267117	1.501253	-0.848344
H	2.295313	0.790219	1.540100
H	1.819294	0.158623	-2.577748
H	-2.333582	0.116789	1.679419
H	2.475540	-1.631125	-0.068167
H	-2.456079	-1.382271	-0.748827
H	-1.575852	2.624612	-0.033469
H	1.615448	2.180371	1.439556
H	2.364611	0.998983	-1.396101
H	-1.818801	-1.235857	2.239402

$2Na^{+}(H_2O)_6$

G = -783.235254 ha

Na	4.772319	1.422324	-1.043188
Na	-4.350315	0.114356	0.140120
O	3.200709	-1.427614	1.491329
H	2.740646	-1.585884	2.323343
H	2.565495	-1.682967	0.797964
O	-3.148815	-1.112525	-1.372373
H	-2.353013	-1.272275	-0.811385
H	-3.482379	-1.986168	-1.600575
O	-3.766887	2.283668	0.649501
H	-4.010741	2.983833	0.034029
H	-3.944844	2.648500	1.523377
O	1.398172	-1.061602	-0.619779
H	1.517174	-1.325643	-1.538741
H	1.795079	-0.172107	-0.527984
O	-1.134445	-1.315446	0.391499
H	-1.130124	-2.157911	0.858152
H	-0.228583	-1.214438	0.029446
O	2.973582	0.996357	0.338572
H	3.154645	0.258985	0.968681
H	2.556067	1.699901	0.847452
